# Supplementary figures and images for: EPHX1 and ERCC2 polymorphisms are associated with cisplatin-induced nephrotoxicity and prognosis in Thai cancer patients
Source: PLoS One. 2025 Jun 17;20(6):e0324699. doi: 10.1371/journal.pone.0324699 (PMC12173183; doi:10.1371/journal.pone.0324699)

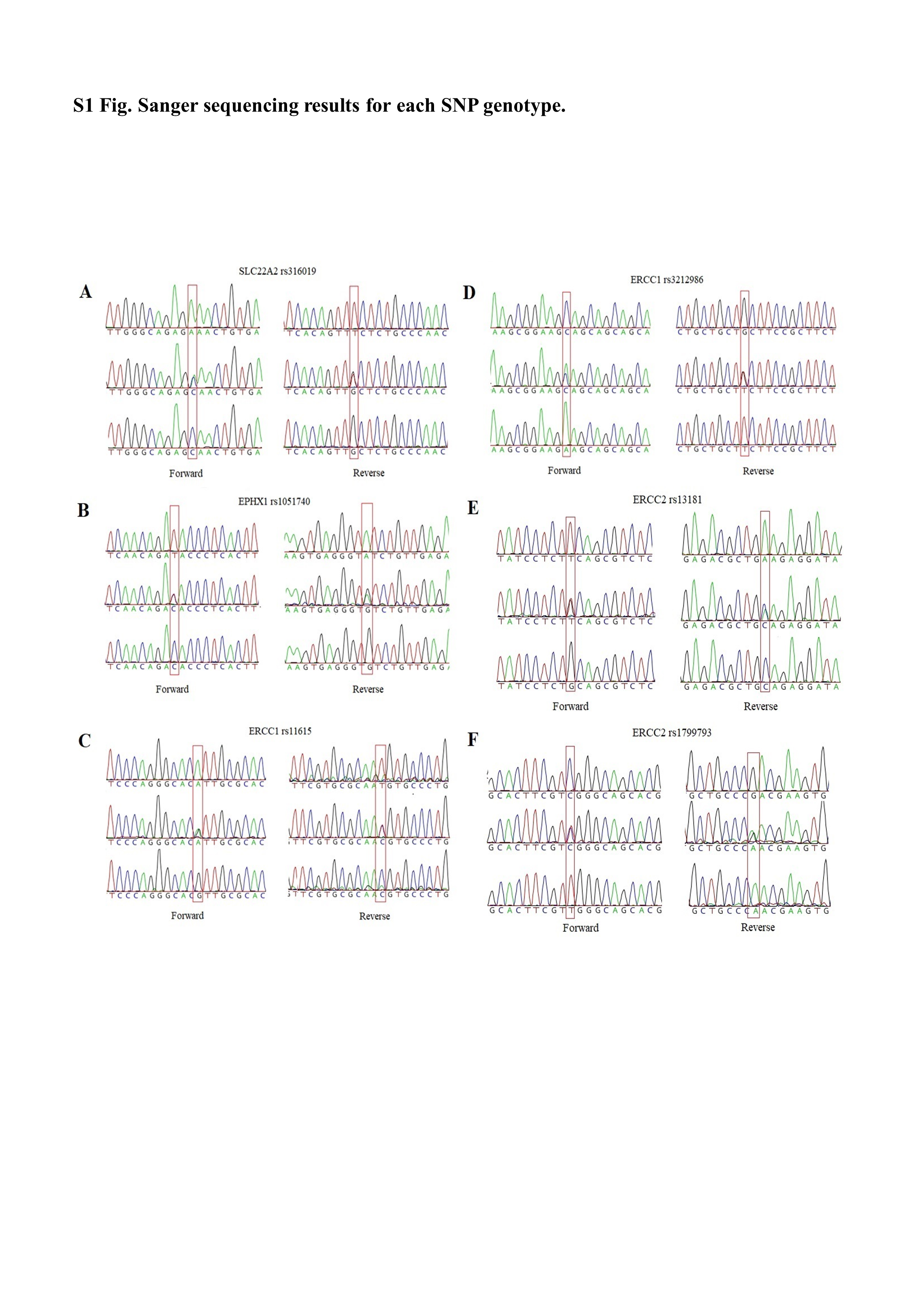

Supplement: S1 Fig — (TIF) [file pone.0324699.s001.TIF]

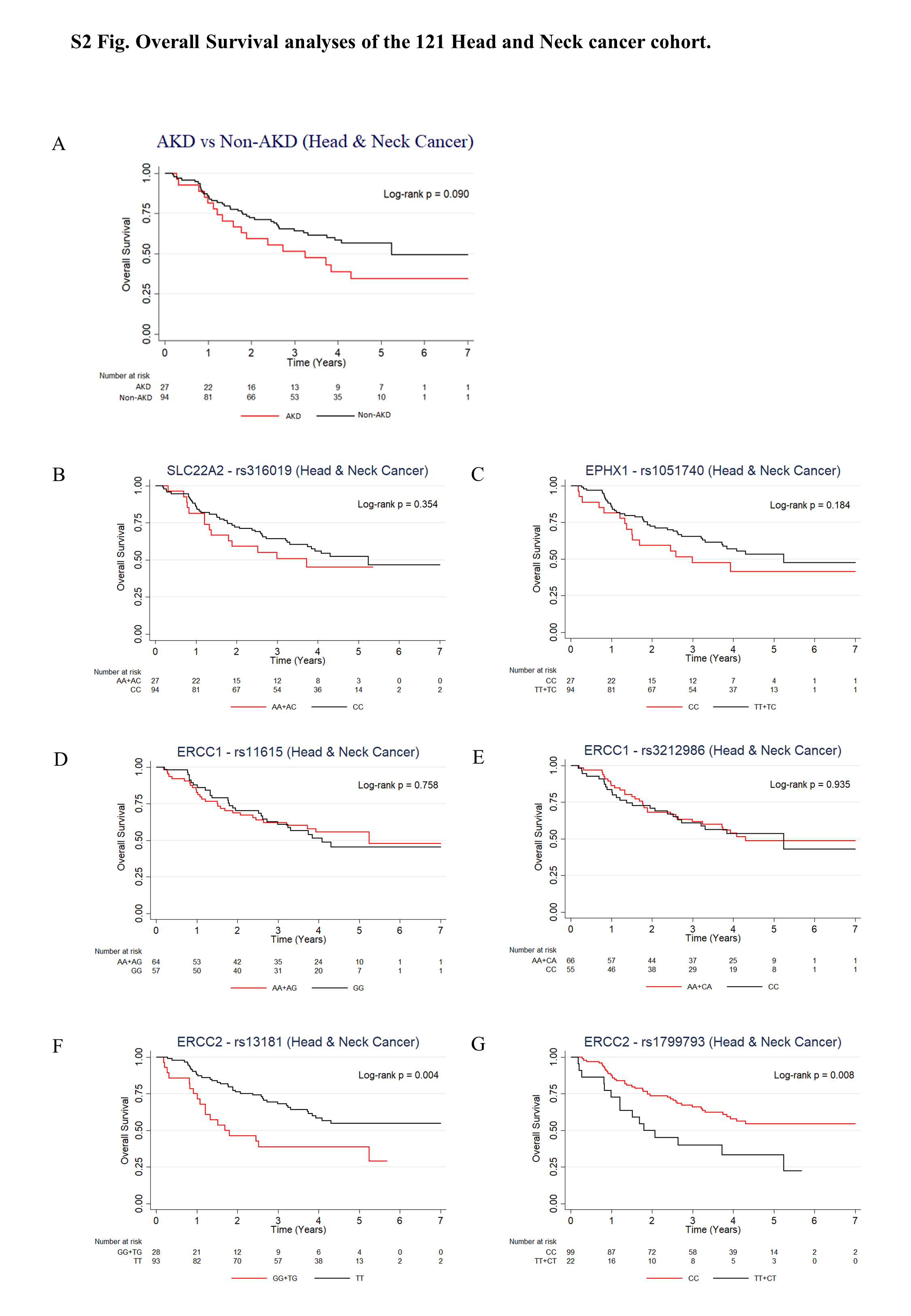

Supplement: S2 Fig — (TIF) [file pone.0324699.s002.TIF]

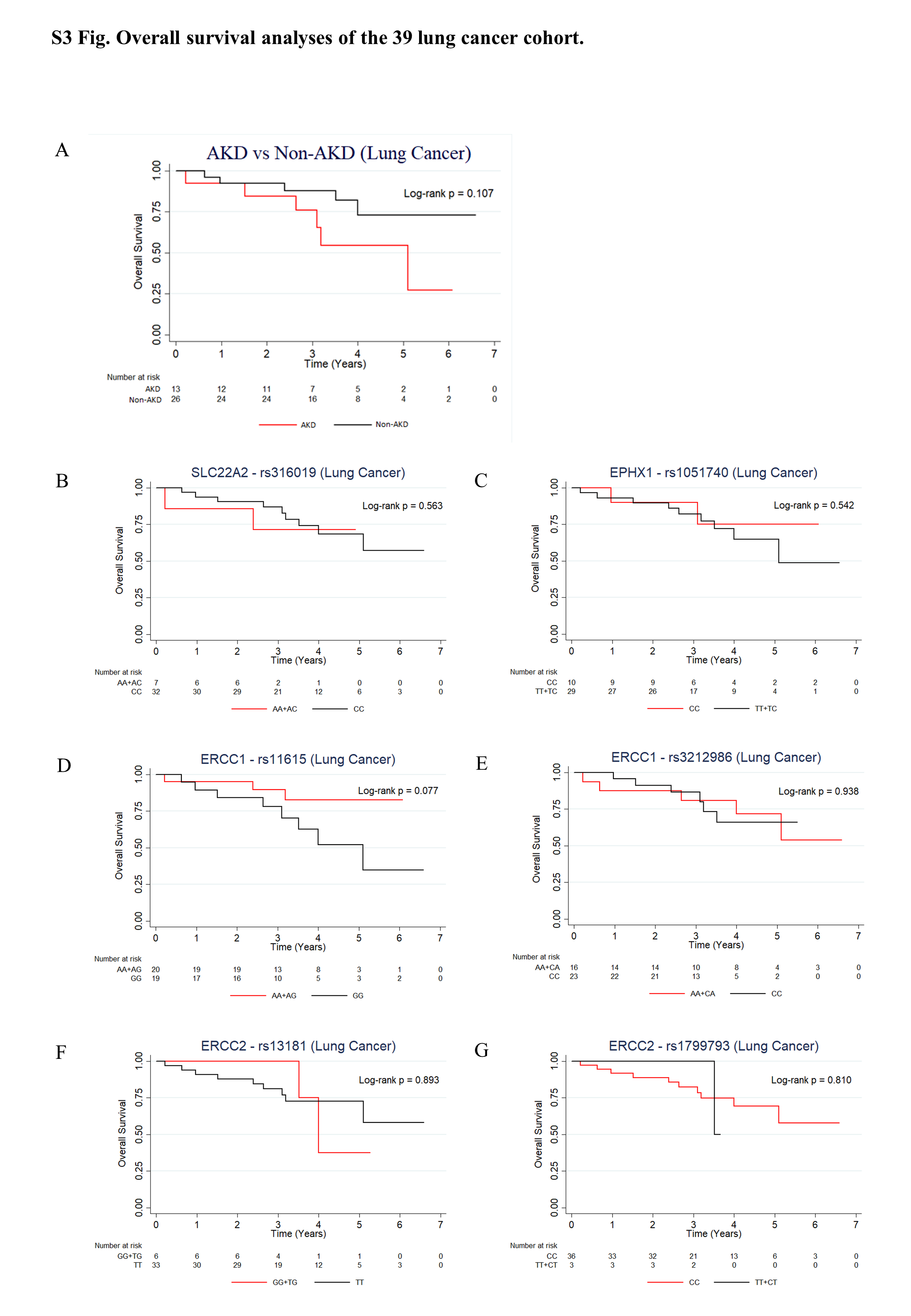

Supplement: S3 Fig — (TIF) [file pone.0324699.s003.TIF]

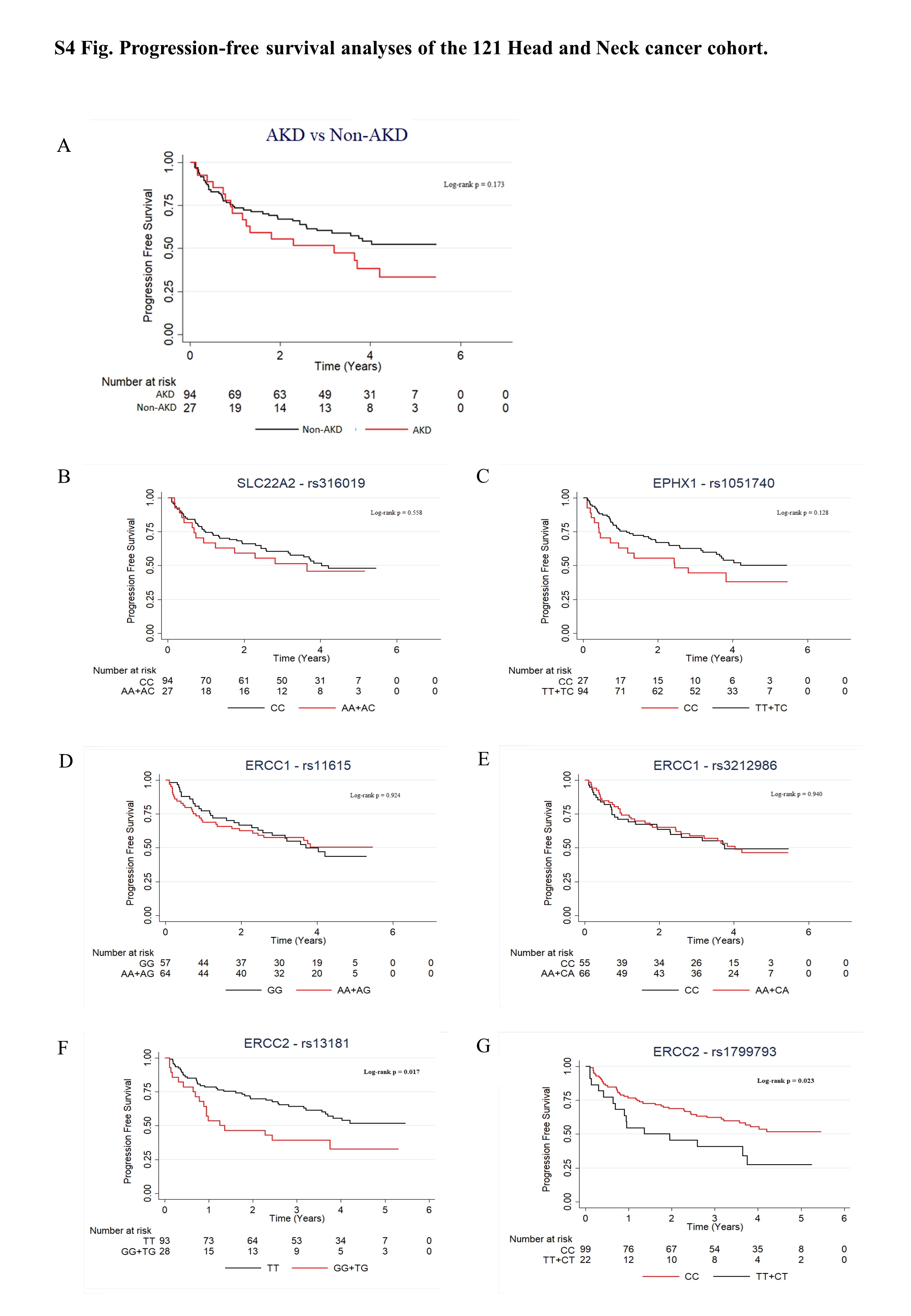

Supplement: S4 Fig — (TIF) [file pone.0324699.s004.TIF]
