# Supplementary material for: EPHX1 and ERCC2 polymorphisms are associated with cisplatin-induced nephrotoxicity and prognosis in Thai cancer patients
Source: PLoS One. 2025 Jun 17;20(6):e0324699. doi: 10.1371/journal.pone.0324699 (PMC12173183; doi:10.1371/journal.pone.0324699)
Supplement: S1 Table — (PDF) [file pone.0324699.s005.pdf]

S1 Table. Primer sequences to perform Sanger sequencing.

Name	Sequence
GREF-rs316019-F	5' GAAGGCAGACTTCTTAGCAGAAT 3'
GREF-rs316019-R	5' GATAGCTGGACAGCCAACTCA 3'
GREF-rs1051740-F	5' TTGCTCTTGTGCTCTGTCCTT 3'
GREF-rs1051740-R	5' AGTCACATTGTGGAAGAAGGCT 3'
GREF-rs11615-F	5' CATGCCCAGAGGCTTCTCAT 3'
GREF-rs11615-R	5' TCAGGGACTGTCCAGGGTTA 3'
GREF-rs3212986-F	5' CAACTCCGGGATCCACCAA 3'
GREF-rs3212986-R	5' TAATAAATCGTCCTCCCAGGCCA 3'
GREF-rs13181-F	5' CTGTGGACGTGACAGTGAGAA 3'
GREF-rs13181-R	5' AACATCCTGTCCCTACTGGC 3'
GREF-rs1799793-F	5' ACTTCACGTACTCCAGCAGC 3'
GREF-rs1799793-R	5' CAAAGAGACAGACGAGCAGC 3'
