# Supplementary material for: EPHX1 and ERCC2 polymorphisms are associated with cisplatin-induced nephrotoxicity and prognosis in Thai cancer patients
Source: PLoS One. 2025 Jun 17;20(6):e0324699. doi: 10.1371/journal.pone.0324699 (PMC12173183; doi:10.1371/journal.pone.0324699)
Supplement: S2 Table — (PDF) [file pone.0324699.s006.pdf]

S2 Table. Touchdown PCR conditions for primer optimization.

No. of Cycles	Denaturation	Annealing	Extension
1	94℃, 2 min	-	-
8	94℃, 15 sec	65℃ – 58℃, 30 sec	72℃, 1 min
27	94℃, 15 sec	58℃, 30 sec	72℃, 1 min
-	-	-	72℃, 7 min
-	-	-	25℃, 1min
