# Supplementary material for: EPHX1 and ERCC2 polymorphisms are associated with cisplatin-induced nephrotoxicity and prognosis in Thai cancer patients
Source: PLoS One. 2025 Jun 17;20(6):e0324699. doi: 10.1371/journal.pone.0324699 (PMC12173183; doi:10.1371/journal.pone.0324699)
Supplement: S3 Table — (PDF) [file pone.0324699.s007.pdf]

S3 Table. Clinical characteristics of AKI and Non-AKI patients after receiving cisplatin chemotherapy

Factors	Overall (n=103)	AKI (n=4) (3.9%)	Non-AKI (n=99) (96.1%)	*P value*
**Sex**				
Male	66 (64.1%)	1 (25.0)	65 (65.7)	0.131
Female	37 (35.9%)	3 (75.0)	34 (34.3)	
**Median Age (range)**	57.3 (31.4 - 73.9)	60.55 (59.4 - 62.8)	56.6 (31.4 – 73.9)	
**Age**				
<65	85 (82.5%)	4 (100)	81 (81.8)	1.000
≥65	18 (17.5%)	0 (0)	18 (18.2)	
**Comorbidity (ICD-10)**				
Hypertension	27 (26.2%)	1 (25.0)	26 (26.3)	1.000
Diabetes Mellitus	8 (7.8%)	0 (0)	8 (8.1)	1.000
Cerebrovascular Disease	7 (6.8%)	0 (0)	7 (7.1)	1.000
Heart Disease	8 (7.8%)	0 (0)	8 (8.1)	1.000
**Cancer Type**				
HNSCC	84 (81.5%)	1 (25.0)	83 (84.0)	**0.020***
Lung	17 (16.5%)	3 (75.0)	14 (14.0)	
Esophagus	2 (2.0%)	0 (0)	2 (2.0)	
**Histology**				
SCC	80 (77.7%)	1 (25.0)	79 (79.8)	**0.034***
Non-SCC	23 (22.3%)	3 (75.0)	20 (20.2)	
**Staging**				
I	9 (8.7%)	1 (25.0)	8 (8.1)	0.234
II	10 (9.7%)	0 (0)	10 (10.1)	
III	23 (24.3%)	2 (50.0)	23 (23.2)	
IV	58 (57.3%)	1 (25.0)	58 (58.6)	
**Baseline mean eGFR (+/-SD) ml/min/1.73m^2**	98.35 ± 15.95	90.80 ± 10.42	98.65 ± 16.09	0.336
**Baseline mean SCr (+/-SD) mg/dL**	0.76 ± 0.18	0.73 ± 0.04	0.76 ± 0.19	0.749

Calculated using Fisher's exact test. * Statistically significant *P* value < 0.05. SCC, Squamous Cell Carcinoma. HNSCC, Head and Neck Squamous Cell Carcinoma
