# Supplementary material for: EPHX1 and ERCC2 polymorphisms are associated with cisplatin-induced nephrotoxicity and prognosis in Thai cancer patients
Source: PLoS One. 2025 Jun 17;20(6):e0324699. doi: 10.1371/journal.pone.0324699 (PMC12173183; doi:10.1371/journal.pone.0324699)
Supplement: S4 Table — (PDF) [file pone.0324699.s008.pdf]

S4 Table. Association of the selected genetic polymorphisms with cisplatin-induced AKD in Head and Neck cancer cohort.

SNP	Model	Genotype	AKD	Non- AKD	Unadjusted OR (95% CI)	*P* value	Adjusted OR (95% CI)	*P* value adj
***SLC22A2* rs316019**	Co-dominant	CC	20 (74.1)	74 (78.7)	1.00		1.00	
		AC	7 (25.9)	17 (18.1)	1.523 (0.555 - 4.180)	0.414	1.523 (0.540 - 4.293)	0.426
		AA	0 (0.0)	3 (3.2)	-	-	-	-
	Dominant	AA+AC	7 (25.9)	20 (21.3)	1.295 (0.480 - 3.493)	0.610	1.306 (0.471 - 3.617)	0.607
	Recessive	CC+AC	27 (100)	91 (96.8)	1.00		1.00	
		AA	0 (0.0)	3 (3.2)	-	-	-	-
	Overdominant	CC+AA	20 (74.1)	77 (81.9)	1.00		1.00	
		AC	7 (25.9)	17 (18.1)	1.585 (0.578 - 4.345)	0.370	1.585 (0.562 - 4.465)	0.383
	Log-Additive				1.062 (0.445 - 2.532)	0.891	1.076 (0.440 - 2.625)	0.872
***EPHX1* rs1051740**	Co-dominant	CC	1 (3.7)	26 (27.7)	1.00		1.00	
		TC	20 (74.1)	39 (41.5)	**13.333 (1.684 - 105.5337)**	**0.014**	17.326 (2.100 - 142.895)	0.008
		TT	6 (22.2)	29 (30.8)	5.379 (0.606 - 47.690)	0.131	5.827 (0.637 - 53.265)	0.118
	Dominant	TT+TC	26 (96.3)	68 (72.3)	**9.941 (1.282 - 77.051)**	**0.028***	**11.794 (1.484 - 93.730)**	**0.020***
	Recessive	CC+TC	21 (77.8)	65 (69.2)	1.00		1.00	
		TT	6 (22.2)	29 (30.8)	0.640 (0.233 - 1.753)	0.386	0.600 (0.211 - 1.703)	0.338
	Overdominant	CC+TT	7 (25.9)	55 (58.5)	1.00		1.00	
		TC	20 (74.1)	39 (41.5)	**4.029 (1.552 - 10.455)**	**0.004***	**5.009 (1.825 - 13.745)**	**0.002***
	Log-Additive				1.357 (0.736 - 2.501)	0.327	1.389 (0.747 - 2.581)	0.298
***ERCC1* rs11615**	Co-dominant	GG	13 (48.2)	44 (46.8)	1.00		1.00	
		AG	11 (40.7)	43 (45.7)	0.865 (0.349 - 2.143)	0.755	0.893 (0.352 - 2.260)	0.811
		AA	3 (11.1)	7 (7.5)	1.450 (0.327 - 6.418)	0.624	1.007 (0.211 - 4.797)	0.992
	Dominant	AA+AG	14 (51.8)	50 (53.2)	0.947 (0.402 - 2.232)	0.902	0.913 (0.378 - 2.202)	0.839
	Recessive	GG+AG	24 (88.9)	87 (92.6)	1.00		1.00	
		AA	3 (11.1)	7 (7.4)	1.553 (0.373 - 6.466)	0.545	1.062 (0.236 - 4.762)	0.937
	Overdominant	GG+AA	16 (59.3)	51 (54.3)	1.00		1.00	
		AG	11 (40.7)	43 (45.7)	0.815 (0.342 - 1.943)	0.645	0.892 (0.365 - 2.178)	0.802
	Log-Additive				1.059 (0.541 - 2.071)	0.867	0.959 (0.486 - 1.891)	0.904

S4 Table, continued

SNP	Model	Genotype	AKD	Non-AKD	Unadjusted OR (95% CI)	*P* value	Adjusted OR (95% CI)	*P* value adj
***ERCC1* rs3212986**	Co-dominant	CC	12 (44.4)	43 (45.7)	1.00		1.00	
		CA	11 (40.7)	42 (44.7)	0.938 (0.373 - 2.359)	0.893	1.050 (0.406 - 2.710)	0.920
		AA	4 (14.8)	9 (9.6)	1.592 (0.416 - 6.084)	0.496	1.696 (0.430 - 6.689)	0.450
	Dominant	AA+AC	15 (55.6)	51 (54.3)	1.053 (0.445 - 2.492)	0.905	1.170 (0.483 - 2.836)	0.727
	Recessive	CC+CA	23 (85.2)	85 (90.4)	1.00		1.00	
		AA	4 (14.8)	9 (9.6)	1.642 (0.463 - 5.817)	0.442	1.657 (0.453 - 6.053)	0.445
	Overdominant	CC+AA	16 (59.3)	52 (55.3)	1.00		1.00	
		CA	11 (40.7)	42 (44.7)	0.851 (0.357 - 2.029)	0.716	0.940 (0.384 - 2.298)	0.893
	Log-Additive				1.157 (0.612 - 2.188)	0.652	1.225 (0.642 - 2.336)	0.537
***ERCC2* rs13181**	Co-dominant	TT	22 (81.5)	71 (75.5)	1.00		1.00	
		TG	5 (18.5)	21 (22.3)	0.768 (0.259 - 2.276)	0.635	0.664 (0.204 - 2.154)	0.496
		GG	0 (0.0)	2 (2.1)	-	-	-	-
	Dominant	GG+TG	5 (18.5)	23 (24.5)	0.701 (0.238 - 2.063)	0.520	0.615 (0.190 - 1.984)	0.416
	Recessive	TT+TG	27 (100)	92 (97.9)	1.00		1.00	
		GG	0 (0.0)	2 (2.1)	-	-	-	-
	Overdominant	TT+GG	22 (81.5)	73 (77.7)	1.00		1.00	
		TG	5 (18.5)	21 (22.3)	0.790 (0.266 - 2.339)	0.670	0.690 (0.213 - 2.226)	0.535
	Log-Additive				0.667 (0.242 - 1.833)	0.433	0.592 (0.195 - 1.798)	0.356
***ERCC2* rs1799793**	Co-dominant	CC	23 (85.2)	76 (80.9)	1.00		1.00	
		CT	3 (11.1)	17 (18.1)	0.583 (0.156 - 2.167)	0.421	0.499 (0.126 - 1.975)	0.323
		TT	1 (3.7)	1 (1.0)	3.304 (0.198 - 54.927)	0.405	4.547 (0.241 - 85.521)	0.312
	Dominant	TT+CT	4 (14.8)	18 (19.1)	0.734 (0.225 - 2.388)	0.608	0.661 (0.192 - 2.270)	0.512
	Recessive	CC+CT	26 (96.3)	93 (98.9)	1.00		1.00	
		TT	1 (3.7)	1 (1.1)	3.576 (0.216 - 59.157)	0.373	5.103 (0.272 - 95.614)	0.276
	Overdominant	CC+TT	24 (88.9)	77 (81.9)	1.00		1.00	
		CT	3 (11.1)	17 (18.1)	0.566 (0.152 - 2.098)	0.395	0.481 (0.122 - 1.888)	0.294
	Log-Additive				0.913 (0.336 - 2.483)	0.860	0.870 (0.301 - 2.516)	0.798

Model was adjusted with age and sex variables. OR, Odds Ratio. 95% CI, 95% Confidence Interval. Adj, adjusted *P* value. * Statistically significant *P* value < 0.05.

S4 Table. Association of the selected genetic polymorphisms with cisplatin-induced AKD in Head and Neck cancer cohort with bootstrap estimation.

SNP	Model	Genotype	AKD	Non- AKD	Unadjusted OR (95% CI)	*P* value	Unadjusted Bootstrap estimation	P value _bootstrap	GOF	Adjusted OR (95% CI)	*P* value adj	Adjusted Bootstrap estimation	P value adj_bootstrap	GOF
SLC22A2 rs316019	**Co-dominant**	CC	20 (74.1)	74 (78.7)	1					1				
		AC	7 (25.9)	17 (18.1)	1.523 (0.555 - 4.180)	0.414	NA	NA		1.524 (0.541 - 4.294)	0.424	NA	NA	0.593
		AA	0 (0.0)	3 (3.2)	-	-				-	-			
	**Dominant**	AA+AC	7 (25.9)	20 (21.3)	1.295 (0.480 - 3.493)	0.610	1.295 (0.418 - 4.002)	0.653	NA	1.307 (0.472- 3.614)	0.606	1.307 (0.417-4.095)	0.646	0.566
	**Recessive**	CC+AC	27 (100)	91 (96.8)	1					1				
		AA	0 (0.0)	3 (3.2)	-	-	NA	NA	NA	-	-	NA	NA	0.657
	**Overdominant**	CC+AA	20 (74.1)	77 (81.9)	1					1				
		AC	7 (25.9)	17 (18.1)	1.585 (0.578 - 4.345)	0.370	1.585 (0.531-4.731)	0.409	NA	1.586 (0.563 - 4.464)	0.382	1.586 (0.492-5.109)	0.439	0.596
	**Log-Additive**				1.062 (0.445 - 2.532)	0.891	1.062 (0.423-2.669)	0.897	0.228	1.077 (0.442 - 2.624)	0.870	1.077 (0.419-2.765)	0.877	0.592
EPHX1 rs1051740	**Co-dominant**	CC	1 (3.7)	26 (27.7)	1					1				
		TC	20 (74.1)	39 (41.5)	**13.333 (1.684 - 105.5337)**	**0.014**	**13.333(4.467-39.790)**	**<0.001**	NA	**17.319 (2.105 - 142.478)**	**0.008**	**17.319 (5.131-58.460)**	**<0.001**	0.571
		TT	6 (22.2)	29 (30.8)	5.379 (0.606 - 47.690)	0.131	**5.379 (1.407-20.562)**	**0.014**		5.824 (0.641 - 52.913)	0.118	**5.824 (1.357-24.985)**	**0.018**	
	**Dominant**	TT+TC	26 (96.3)	68 (72.3)	**9.941 (1.282 - 77.051)**	**0.028***	**9.941(3.477-28.418)**	**<0.001**	NA	**11.702 (1.478 - 92.628)**	**0.020***	**11.702 (3.629-37.730)**	**<0.001**	0.569
	**Recessive**	CC+TC	21 (77.8)	65 (69.2)	1					1				
		TT	6 (22.2)	29 (30.8)	0.640 (0.233 - 1.753)	0.386	0.640 (0.214-1.914)	0.425	NA	0.604 (0.214- 1.707)	0.342	0.604 (0.191-1.913)	0.392	0.554
	**Overdominant**	CC+TT	7 (25.9)	55 (58.5)	1					1				
		TC	20 (74.1)	39 (41.5)	**4.029 (1.552 - 10.455)**	**0.004***	**4.029 (1.454-11.160)**	**0.007**	NA	**5.005 (1.822 - 13.748)**	**0.002***	**5.005 (1.632-15.346)**	**0.005**	0.616
	**Log-Additive**				1.357 (0.736 - 2.501)	0.327	1.357 (0.853-2.159)	0.197	0.0013	1.392 (0.748 - 2.590)	0.296	1.392 (0.853-2.272)	0.185	0.427
ERCC1 rs11615	**Co-dominant**	GG	13 (48.2)	44 (46.8)	1					1				
		AG	11 (40.7)	43 (45.7)	0.865 (0.349 - 2.143)	0.755	0.865 (0.340-2.202)	0.762	NA	0.895 (0.354 - 2.259)	0.815	0.895 (0.308-2.597)	0.839	0.605
		AA	3 (11.1)	7 (7.5)	1.450 (0.327 - 6.418)	0.624	1.450 (0.314-6.688)	0.633		1.010 (0.213 - 4.793)	0.990	1.010 (0.188-5.424)	0.990	
	**Dominant**	AA+AG	14 (51.8)	50 (53.2)	0.947 (0.402 - 2.232)	0.902	0.947(0.389-2.306)	0.906	NA	0.915 (0.380 - 2.201)	0.844	0.915 (0.351-2.382)	0.856	0.630
	**Recessive**	GG+AG	24 (88.9)	87 (92.6)	1					1				
		AA	3 (11.1)	7 (7.4)	1.553 (0.373 - 6.466)	0.545	1.553 (0.341-7.067)	0.569	NA	1.063 (0.237 - 4.758)	0.936	1.063 (0.227-4.964)	0.938	0.634
	**Overdominant**	GG+AA	16 (59.3)	51 (54.3)	1					1				
		AG	11 (40.7)	43 (45.7)	0.815 (0.342 - 1.943)	0.645	0.815 (0.315-2.104)	0.673	NA	0.894 (0.366 - 2.178)	0.805	0.894 (0.348-2.292)	0.816	0.605
	**Log-Additive**				1.059 (0.541 - 2.071)	0.867	1.059 (0.498-2.248)	0.881	0.514	0.960 (0.488 - 1.889)	0.908	0.960 (0.469-1.966)	0.913	0.624

S4 Table, continued

SNP	Model	Genotype	AKD	Non- AKD	Unadjusted OR (95% CI)	*P* value	Unadjusted Bootstrap estimation	P value _bootstrap	GOF	Adjusted OR (95% CI)	*P* value adj	Adjusted Bootstrap estimation	P value adj_bootstrap	GOF
ERCC1 rs3212986	**Co-dominant**	CC	12 (44.4)	43 (45.7)	1					1				
		CA	11 (40.7)	42 (44.7)	0.938 (0.373 - 2.359)	0.893	0.938 (0.338-2.600)	0.903	NA	1.051 (0.407 - 2.712)	0.918	1.051 (0.385-2.864)	0.922	
		AA	4 (14.8)	9 (9.6)	1.592 (0.416 - 6.084)	0.496	1.592 (0.360-7.031)	0.539		1.692 (0.429 - 6.670)	0.452	1.692 (0.362-7.895)	0.503	0.585
	**Dominant**	AA+AC	15 (55.6)	51 (54.3)	1.053 (0.445 - 2.492)	0.905	1.053 (0.433-2.563)	0.908	NA	1.171 (0.483 - 2.836)	0.726	1.171 (0.451-3.036)	0.745	0.599
	**Recessive**	CC+CA	23 (85.2)	85 (90.4)	1					1				
		AA	4 (14.8)	9 (9.6)	1.642 (0.463 - 5.817)	0.442	1.642 (0.440-6.128)	0.460	NA	1.653 (0.453 - 6.028)	0.446	1.653 (0.389-7.019)	0.496	0.6707
	**Overdominant**	CC+AA	16 (59.3)	52 (55.3)	1					1				
		CA	11 (40.7)	42 (44.7)	0.851 (0.357 - 2.029)	0.716	0.851 (0.345-2.099)	0.726	NA	0.941 (0.385 - 2.299)	0.895	0.941 (0.353 -2.505)	0.904	0.541
	**Log-Additive**				1.157 (0.612 - 2.188)	0.652	1.157 (0.555-2.413)	0.696	0.537	1.225 (0.642 - 2.335)	0.537	1.225 (0.591 - 2.537)	0.585	0.620
ERCC2 rs13181	**Co-dominant**	TT	22 (81.5)	71 (75.5)	1					1				
		TG	5 (18.5)	21 (22.3)	0.768 (0.259 - 2.276)	0.635	NA	NA		0.664 (0.204 - 2.154)	0.496	NA	NA	0.658
		GG	0 (0.0)	2 (2.1)	-	-				-	-			
	**Dominant**	GG+TG	5 (18.5)	23 (24.5)	0.701 (0.238 - 2.063)	0.520	0.701 (0.205-2.394)	0.571	NA	0.644 (0.210 - 1.975)	0.442	0.644 (0.202-2.049)	0.457	0.675
	**Recessive**	TT+TG	27 (100)	92 (97.9)	1					1				
		GG	0 (0.0)	2 (2.1)	-	-	NA	NA	NA	-	-	NA	NA	0.632
	**Overdominant**	TT+GG	22 (81.5)	73 (77.7)	1					1				
		TG	5 (18.5)	21 (22.3)	0.790 (0.266 - 2.339)	0.670	0.790 (0.243-2.558)	0.694	NA	0.714 (0.231 - 2.201)	0.558	0.714 (0.217 -2.343)	0.579	0.658
	**Log-Additive**				0.667 (0.242 - 1.833)	0.433	0.667 (0.224-1.983)	0.467	0.555	0.621 (0.215 - 1.797)	0.380	0.621 (0.223-1.728)	0.362	0.686
ERCC2 rs1799793	**Co-dominant**	CC	23 (85.2)	76 (80.9)	1					1				
		CT	3 (11.1)	17 (18.1)	0.583 (0.156 - 2.167)	0.421	0.583 (0.159-2.128)	0.414	NA	0.510 (0.131 - 1.974)	0.330	0.510 (0.137-1.892)	0.314	
		TT	1 (3.7)	1 (1.0)	3.304 (0.198 - 54.927)	0.405	3.304 (0.885-12.336)	0.075		4.673 (0.249 - 87.401)	0.302	**4.673 (1.453-15.029)**	**0.010**	0.611
	**Dominant**	TT+CT	4 (14.8)	18 (19.1)	0.734 (0.225 - 2.388)	0.608	0.734 (0.206-2.617)	0.634	NA	0.675 (0.200 - 2.271)	0.526	0.675 (0.191-2.377)	0.541	0.633
	**Recessive**	CC+CT	26 (96.3)	93 (98.9)	1					1				
		TT	1 (3.7)	1 (1.1)	3.576 (0.216 - 59.157)	0.373	3.576 (0.944-13.546)	0.061	NA	5.091 (0.275 - 94.058)	0.274	**5.091 (1.597 - 16.223)**	**0.006**	0.602
	**Overdominant**	CC+TT	24 (88.9)	77 (81.9)	1					1				
		CT	3 (11.1)	17 (18.1)	0.566 (0.152 - 2.098)	0.395	0.566 (0.149-2.142)	0.402	NA	0.495 (0.128 - 1.904)	0.307	0.495 (0.133-1.838)	0.294	0.647
	**Log-Additive**				0.913 (0.336 - 2.483)	0.860	0.913 (0.275-3.029)	0.883	0.200	0.879 (0.309 - 2.496)	0.810	0.879 (0.233-3.319)	0.850	0.6229

Model was adjusted with age and sex variables. OR, Odds Ratio. 95% CI, 95% Confidence Interval. Adj, adjusted *P* value. * Statistically significant *P* value < 0.05. GOF, Goodness-of-Fit
