# Supplementary material for: EPHX1 and ERCC2 polymorphisms are associated with cisplatin-induced nephrotoxicity and prognosis in Thai cancer patients
Source: PLoS One. 2025 Jun 17;20(6):e0324699. doi: 10.1371/journal.pone.0324699 (PMC12173183; doi:10.1371/journal.pone.0324699)
Supplement: S5 Table — (PDF) [file pone.0324699.s009.pdf]

S5 Table. Association of the selected genetic polymorphisms with cisplatin-induced AKD in Head and Neck and Esophageal cancer cohort.

SNP	Model	Genotype	AKD	Non-AKD	Unadjusted OR (95% CI)	*P* value	Adjusted OR (95% CI)	*P* value adj
***SLC22A2* rs316019**	Co-dominant	CC	22 (73.3)	79 (79.0)	1.00		1.00	
		AC	8 (26.7)	18 (18.0)	1.595 (0.612 - 4.157)	0.339	1.529 (0.571 - 4.096)	0.398
		AA	0 (0.0)	3 (3.0)	-	-	-	-
	Dominant	AA+AC	8 (26.7)	21 (21.0)	1.367 (0.533 - 3.507)	0.514	1.329 (0.504 - 3.498)	0.565
	Recessive	CC+AC	30 (100)	97 (97.0)	1.00		1.00	
		AA	0 (0.0)	3 (3.0)	-	-	-	-
	Overdominant	CC+AA	22 (73.3)	82 (82.0)	1.00		1.00	
		AC	8 (26.7)	18 (18.0)	1.656 (0.636 - 4.311)	0.301	1.585 (0.592 - 4.243)	0.359
	Log-Additive				1.118 (0.489 - 2.558)	0.790	1.106 (0.471 - 2.597)	0.817
***EPHX1* rs1051740**	Co-dominant	CC	2 (6.7)	27 (27.0)	1.00		1.00	
		TC	22 (73.3)	42 (42.0)	7.071 (1.537 - 32.531)	0.012	8.750 (1.822 - 42.003)	0.007
		TT	6 (20.0)	31 (31.0)	2.612 (0.486 - 14.039)	0.263	2.662 (0.479 - 14.782)	0.263
	Dominant	TT+TC	28 (93.3)	73 (73.0)	**5.178 (1.154 - 23.228)**	**0.032***	**5.850 (1.271 - 26.916)**	**0.023***
	Recessive	CC+TC	24 (80.0)	69 (69.0)	1.00		1.00	
		TT	6 (20.0)	31 (31.0)	0.556 (0.206 - 1.497)	0.246	0.505 (0.181 - 1.411)	0.193
	Overdominant	CC+TT	8 (26.7)	58 (58.0)	1.00		1.00	
		TC	22 (73.3)	42 (42.0)	**3.797 (1.541 - 9.353)**	**0.004***	**4.666 (1.797 - 12.118)**	**0.002***
	Log-Additive				1.204 (0.675 - 2.149)	0.528	1.211 (0.670 - 2.188)	0.525
***ERCC1* rs11615**	Co-dominant	GG	16 (53.3)	45 (45.0)	1.00		1.00	
		AG	11 (36.7)	47 (47.0)	0.658 (0.275 - 1.570)	0.346	0.686 (0.280 - 1.678)	0.409
		AA	3 (10.0)	8 (8.0)	1.054 (0.248 - 4.470)	0.942	0.779 (0.171 - 3.543)	0.747
	Dominant	AA+AG	14 (46.7)	55 (55.0)	0.715 (0.315 - 1.622)	0.423	0.702 (0.301 - 1.636)	0.413
	Recessive	GG+AG	27 (90.0)	92 (92.0)	1.00		1.00	
		AA	3 (10.0)	8 (8.0)	1.277 (0.316 - 5.153)	0.730	0.925 (0.214 - 3.991)	0.917
	Overdominant	GG+AA	19 (63.3)	53 (53.0)	1.00		1.00	
		AG	11 (36.7)	47 (47.0)	0.652 (0.281 - 1.512)	0.320	0.714 (0.301 - 1.693)	0.445
	Log-Additive				0.853 (0.444 - 1.637)	0.633	0.796 (0.411 - 1.542)	0.500

S5 Table, continued

SNP	Model	Genotype	AKD	Non-AKD	Unadjusted OR (95% CI)	*P* value	Adjusted OR (95% CI)	*P* value adj
***ERCC1* rs3212986**	Co-dominant	CC	13 (43.3)	46 (46.0)	1.00		1.00	
		CA	12 (40.0)	45 (45.0)	0.943 (0.389 - 2.287)	0.898	1.040 (0.418 - 2.591)	0.931
		AA	5 (16.7)	9 (9.0)	1.965 (0.560 - 6.893)	0.291	2.112 (0.581 - 7.671)	0.256
	Dominant	AA+AC	17 (56.7)	54 (54.0)	1.113 (0.489 - 2.534)	0.797	1.226 (0.526 - 2.861)	0.636
	Recessive	CC+CA	25 (83.3)	91 (91.0)	1.00		1.00	
		AA	5 (16.7)	9 (9.00)	2.022 (0.621 - 6.577)	0.242	2.071 (0.616 - 6.967)	0.239
	Overdominant	CC+AA	18 (60.0)	55 (55.0)	1.00		1.00	
		CA	12 (40.0)	45 (45.0)	0.814 (0.355 - 1.868)	0.629	0.885 (0.376 - 2.079)	0.780
	Log-Additive				1.258 (0.687 - 2.304)	0.456	1.329 (0.718 - 2.462)	0.365
***ERCC2* rs13181**	Co-dominant	TT	24 (80.0)	75 (75.0)	1.00		1.00	
		TG	6 (20.0)	23 (23.0)	0.815 (0.297 - 2.236)	0.692	0.712 (0.241 - 2.101)	0.539
		GG	0 (0.0)	2 (2.0)	-	-	-	-
	Dominant	GG+TG	6 (20.0)	25 (25.0)	0.75 (0.275 - 2.043)	0.574	0.666 (0.226 - 1.955)	0.460
	Recessive	TT+TG	30 (100)	98 (98.0)	1.00		1.00	
		GG	0 (0.0)	2 (2.0)	-	-	-	-
	Overdominant	TT+GG	24 (80.0)	77 (77.0)	1.00		1.00	
		TG	6 (20.0)	23 (23.0)	0.836 (0.305 - 2.294)	0.729	0.734 (0.249 - 2.160)	0.575
	Log-Additive				0.710 (0.277 - 1.819)	0.476	0.640 (0.230 - 1.783)	0.394
***ERCC2* rs1799793**	Co-dominant	CC	26 (86.7)	80 (80.0)	1.00		1.00	
		CT	3 (10.0)	19 (19.0)	0.485 (0.132 - 1.774)	0.275	0.438 (0.113 - 1.696)	0.232
		TT	1 (3.3)	1 (1.0)	3.076 (0.185 - 50.949)	0.433	4.458 (0.234 - 84.752)	0.320
	Dominant	TT+CT	4 (13.3)	20 20.0)	0.615 (0.192 - 1.965)	0.412	0.579 (0.172 - 1.952)	0.379
	Recessive	CC+CT	29 (96.7)	99 (99.0)	1.00		1.00	
		TT	1 (3.3)	1 (1.0)	3.413 (0.207 - 56.280)	0.391	5.150 (0.270 - 98.052)	0.276
	Overdominant	CC+TT	27 (90.0)	81 (81.0)	1.00		1.00	
		CT	3 (10.0)	19 (19.0)	0.473 (0.129 - 1.726)	0.257	0.422 (0.109 - 1.626)	0.210
	Log-Additive				0.785 (0.290 - 2.126)	0.635	0.773 (0.269 - 2.226)	0.635

Model was adjusted with age and sex variables. OR, Odds Ratio. 95% CI, 95% Confidence Interval. Adj, adjusted *P* value. * Statistically significant *P* value < 0.05.
