# Supplementary material for: EPHX1 and ERCC2 polymorphisms are associated with cisplatin-induced nephrotoxicity and prognosis in Thai cancer patients
Source: PLoS One. 2025 Jun 17;20(6):e0324699. doi: 10.1371/journal.pone.0324699 (PMC12173183; doi:10.1371/journal.pone.0324699)
Supplement: S6 Table — (PDF) [file pone.0324699.s010.pdf]

S6 Table. Association of the selected genetic polymorphisms with cisplatin-induced AKD in lung cancer cohort.

SNP	Model	Genotype	AKD	Non-AKD	Unadjusted OR (95% CI)	*P* value	Adjusted OR (95% CI)	*P* value adj
***SLC22A2* rs316019**	Co-dominant	CC	10 (76.9)	22 (84.6)	1.00		1.00	
		AC	3 (23.1)	4 (15.4)	1.65 (0.309 - 8.793)	0.557	1.363 (0.240 - 7.728)	0.726
		AA	0 (0.0)	0 (0.0)	-	-	-	-
	Dominant	AA+AC	3 (23.1)	4 (15.4)	1.65 (0.309 - 8.793)	0.557	1.363 (0.240 - 7.728)	0.726
	Recessive	CC+AC	13 (100)	26 (100)	1.00		1.00	
		AA	0 (0.0)	0 (0.0)	-	-	-	-
	Overdominant	CC+AA	10 (76.9)	22 (84.6)	1.00		1.00	
		AC	3 (23.1)	4 (15.4)	1.65 (0.309 - 8.793)	0.557	1.363 (0.240 - 7.728)	0.726
	Log-Additive				1.65 (0.309 - 8.793)	0.557	1.363 (0.240 - 7.728)	0.726
***EPHX1* rs1051740**	Co-dominant	CC	4 (30.8)	6 (23.1)	1.00		1.00	
		TC	8 (61.5)	15 (57.7)	0.8 (0.173 - 3.689)	0.775	0.960 (0.186 - 4.953)	0.962
		TT	1 (7.7)	5 (19.2)	0.300 (0.024 - 3.625)	0.344	0.307 (0.023 - 3.953)	0.365
	Dominant	TT+TC	9 (69.2)	20 (76.9)	0.675 (0.152 - 2.994)	0.605	0.765 (0.158 - 3.704)	0.740
	Recessive	CC+TC	12 (92.3)	21 (80.8)	1.00		1.00	
		TT	1 (7.7)	5 (19.2)	0.350 (0.036 - 3.357)	0.363	0.315 (0.030 - 3.211)	0.330
	Overdominant	CC+TT	5 (38.5)	11 (42.3)	1.00		1.00	
		TC	8 (61.5)	15 (57.7)	1.173 (0.300 - 4.578)	0.818	1.411 (0.327 - 6.072)	0.644
	Log-Additive				0.611 (0.206 - 1.811)	0.375	0.636 (0.208 - 1.937)	0.426
***ERCC1* rs11615**	Co-dominant	GG	5 (38.5)	14 (53.8)	1.00		1.00	
		AG	6 (46.1)	10 (38.5)	1.68 (0.398 - 7.074)	0.479	1.495 (0.336 - 6.644)	0.597
		AA	2 (15.4)	2 (7.7)	2.8 (0.307 - 25.524)	0.361	2.421 (0.243 - 24.095)	0.451
	Dominant	AA+AG	8 (61.5)	12 (46.2)	1.866 (0.480 - 7.255)	0.368	1.651 (0.403 - 6.762)	0.486
	Recessive	GG+AG	11 (84.6)	24 (92.3)	1.00		1.00	
		AA	2 (15.4)	2 (7.7)	2.181 (0.270 - 17.566)	0.464	1.985 (0.226 - 17.440)	0.536
	Overdominant	GG+AA	7 (53.8)	16 (61.5)	1.00		1.00	
		AG	6 (46.2)	10 (38.5)	1.371 (0.356 - 5.272)	0.646	1.248 (0.307 - 5.067)	0.756
	Log-Additive				1.675 (0.615 - 4.560)	0.312	1.536 (0.542 - 4.354)	0.419

S6 Table, continued

SNP	Model	Genotype	AKD	Non-AKD	Unadjusted OR (95% CI)	*P* value	Adjusted OR (95% CI)	*P* value
***ERCC1* rs3212986**	Co-dominant	CC	7 (53.8)	16 (61.5)	1.00		1.00	
		CA	5 (38.5)	4 (15.4)	2.857 (0.584 - 13.964)	0.195	2.364 (0.404 - 13.817)	0.340
		AA	1 (7.7)	6 (23.1)	0.380 (0.038 - 3.783)	0.410	0.361 (0.028 - 4.530)	0.430
	Dominant	AA+AC	6 (46.2)	10 (38.5)	1.371 (0.356 - 5.272)	0.646	1.210 (0.272 - 5.373)	0.802
	Recessive	CC+CA	12 (92.3)	20 (76.9)	1.00		1.00	
		AA	1 (7.7)	6 (23.1)	0.277 (0.029 - 2.595)	0.261	0.290 (0.024 - 3.401)	0.325
	Overdominant	CC+AA	8 (61.5)	22 (84.6)	1.00		1.00	
		CA	5 (38.5)	4 (15.4)	3.437 (0.734 - 16.096)	0.117	2.783 (0.492 - 15.713)	0.246
	Log-Additive				0.878 (0.366 - 2.103)	0.770	0.846 (0.313 - 2.284)	0.742
***ERCC2* rs13181**	Co-dominant	TT	13 (100)	20 (76.9)	1.00		1.000	
		TG	0 (0.0)	6 (23.1)	-	-	-	-
		GG	0 (0.0)	0 (0.0)	-	-	-	-
	Dominant	GG+TG	0 (0.0)	6 (23.1)	-	-	-	-
	Recessive	TT+TG	13 (100)	26 (100)	1.00	-	1.00	
		GG	0 (0.0)	0 (0.0)	-	-	-	-
	Overdominant	TT+GG	13 (100)	20 (76.9)	1.00		1.00	
		TG	0 (0.0)	6 (23.1)	-	-	-	-
	Log-Additive				-	-	-	-
***ERCC2* rs1799793**	Co-dominant	CC	13 (100)	23 (88.5)	1.00		1.00	
		CT	0 (0.0)	3 (11.5)	-	-		-
		TT	0 (0.0)	0 (0.0)	-	-		-
	Dominant	TT+CT	0 (0.0)	3 (11.5)	-	-		-
	Recessive	CC+CT	13 (100)	26 (100)	1.00		1.00	
		TT	0 (0.0)	0 (0.0)	-	-	-	-
	Overdominant	CC+TT	13 (100)	23 (88.5)	1.00		1.00	
		CT	0 (0.0)	3 (11.5)	-	-	-	-
	Log-Additive				-	-	-	-

Model was adjusted with age and sex variables. OR, Odds Ratio. 95% CI, 95% Confidence Interval. Adj, adjusted *P* value. * Statistically significant *P* value < 0.05.
