# Supplementary material for: EPHX1 and ERCC2 polymorphisms are associated with cisplatin-induced nephrotoxicity and prognosis in Thai cancer patients
Source: PLoS One. 2025 Jun 17;20(6):e0324699. doi: 10.1371/journal.pone.0324699 (PMC12173183; doi:10.1371/journal.pone.0324699)
Supplement: S7 Table — (PDF) [file pone.0324699.s011.pdf]

S7 Table. Associated Risk Factors of Time-To-AKD in this cohort.

Factors	AKD (n=43) (25.4%)	Non-AKD (n=126) (74.6%)	Univariate Cox regression HR (95% CI), *P* value	Multivariate Cox regression HR (95% CI), *P* value
Male	25 (58.1)	85 (67.5)	0.711 (0.387 – 1.303), 0.270	-
Age	-	-	**1.040 (1.006 – 1.076), 0.019**	0.995 (0.950 – 1.042), 0.855
**Age group**				
<65	34 (79.1)	105 (83.3)	0.752 (0.361 – 1.570), 0.449	
**Comorbidity (ICD-10)**				
Hypertension	17 (39.5)	33 (26.2)	1.630 (0.884 – 3.005), 0.117	-
Diabetes Mellitus	4 (9.3)	11 (8.7)	0.992 (0.354 – 2.779), 0.989	-
Cerebrovascular Disease	4 (9.3)	9 (7.1)	1.330 (0.474 – 3.729), 0.588	-
Heart Disease	2 (4.7)	7 (5.6)	0.828 (0.200 – 3.426), 0.795	-
**Histology**				
Non-SCC	14 (32.6)	31 (24.6)	1.394 (0.736 – 2.639), 0.307	-
**Staging Group**				
3 – 4	34 (25.4)	100 (74.6)	0.942 (0.451 – 1.964), 0.874	-
**Baseline mean eGFR (ml/min/1.73m^2)**	-	-	**0.954 (0.934 – 0.974), <0.001**	0.947 (0.910 – 0.985), 0.008
**Baseline mean SCr (mg/dL)**	-	-	**14.490 (2.167 – 96.873), 0.006**	0.368 (0.174 – 7.796), 0.522
**rs316019**				
AC	11 (25.6)	22 (17.5)	1.450 (0.730 – 2.878), 0.288	-
AA	0	3 (2.4)	-	-
**rs1051740**				
TC	30 (69.8)	57 (45.2)	**2.433 (1.012 – 5.847), 0.047**	2.289 (0.947 – 5.535), 0.066
TT	7 (16.2)	36 (28.6)	0.997 (0.335 – 2.971), 0.997	0.988 (0.331 – 2.949), 0.984
**rs11615**				
AG	17 (39.5)	57 (45.2)	0.892 (0.470 – 1.692), 0.727	-
AA	5 (11.6)	10 (8.0)	1.365 (0.514 – 3.624), 0.531	-
**rs3212986**				
CA	17 (39.5)	49 (38.8)	1.077 (0.564 – 2.057), 0.822	-
AA	6 (14.0)	15 (12.0)	1.245 (0.499 – 3.105), 0.638	-
**rs13181**				
TG	6 (14.0)	29 (23.0)	0.558 (0.235 – 1.323), 0.186	-
GG	0	2 (1.6)	-	-
**rs1799793**				
CT	3 (7.0)	22 (17.5)	0.374 (0.115 – 1.212), 0.101	-
TT	1 (2.3)	1 (0.8)	3.820 (0.514 – 28.372), 0.190	-

HR, Hazard Ratio. 95% CI, 95% Confidence Interval. Statistically significant *P* value < 0.05.
