# Supplementary material for: EPHX1 and ERCC2 polymorphisms are associated with cisplatin-induced nephrotoxicity and prognosis in Thai cancer patients
Source: PLoS One. 2025 Jun 17;20(6):e0324699. doi: 10.1371/journal.pone.0324699 (PMC12173183; doi:10.1371/journal.pone.0324699)
Supplement: S8 Table — (PDF) [file pone.0324699.s012.pdf]

S8 Table. Risk Factors of Overall Survival.

Factors	Alive (n=94) (55.6%)	Death (n=75) (44.4%)	Univariate Cox regression HR (95% CI), *P* value	Multivariate Cox regression HR (95% CI), *P* value
Male	54 (49.1)	56 (50.9)	**1.815 (1.077 – 3.058), 0.025**	1.455 (0.822 – 2.574), 0.197
Median Age (range)	-	-	1.021 (0.996 – 1.046), 0.088	-
**Age group**				
<65	76 (54.7)	63 (45.3)	0.933 (0.503 – 1.732), 0.828	-
**Comorbidity (ICD-10)**				
Hypertension	29 (58.0)	21 (42.0)	0.912 (0.551 – 1.511), 0.723	-
Diabetes Mellitus	5 (33.3)	10 (66.7)	**2.219 (1.133 – 4.343), 0.020**	1.687 (0.837 – 3.401), 0.143
Cerebrovascular Disease	7 (53.9)	6 (46.2)	1.289 (0.559 – 2.970), 0.551	-
Heart Disease	2 (22.2)	7 (77.8)	**2.674 (1.222 – 5.851), 0.014**	1.780 (0.788 – 4.023), 0.165
**Histology**				
Non-SCC	32 (71.1)	13 (28.9)	**0.508 (0.279 – 0.925), 0.027**	0.649 (0.339 – 1.242), 0.192
**Staging Group**				
3 – 4	69 (51.5)	65 (48.5)	1.882 (0.967 – 3.665), 0.063	-
**Baseline mean eGFR (ml/min/1.73m^2)**	-	-	0.998 (0.982 – 1.014), 0.849	-
**Baseline mean SCr (mg/dL)**	-	-	1.281 (0.314 – 5.213), 0.729	-
**rs316019 A Carriage**	18 (50.0)	18 (50.0)	1.386 (0.815 – 2.356), 0.228	-
**rs1051740 T Carriage**	73 (56.2)	57 (43.8)	0.883 (0.520 – 1.502), 0.649	-
**rs11615 A Carriage**	53 (59.6)	36 (40.4)	0.845 (0.537 – 1.330), 0.468	-
**rs3212986 A Carriage**	46 (52.9)	41 (47.1)	1.117 (0.708 – 1.760), 0.633	-
**rs13181 G Carriage**	14 (37.8)	23 (62.2)	**2.080 (1.270 – 3.407), 0.004**	1.837 (0.918 – 3.674), 0.086
**rs1799793 T Carriage**	9 (33.3)	18 (66.7)	**2.107 (1.238 – 3.586), 0.006**	1.029 (0.477 – 2.220), 0.940

HR, Hazard Ratio. 95% CI, 95% Confidence Interval. Statistically significant *P* value < 0.05.
