# Supplementary material for: EPHX1 and ERCC2 polymorphisms are associated with cisplatin-induced nephrotoxicity and prognosis in Thai cancer patients
Source: PLoS One. 2025 Jun 17;20(6):e0324699. doi: 10.1371/journal.pone.0324699 (PMC12173183; doi:10.1371/journal.pone.0324699)
Supplement: Attachment — Submitted filename: Response to Reviewers.docx [file pone.0324699.s014.docx]

Dear the Editor in Chief of PLOS ONE,

Thank you for your kind consideration of our manuscript entitled “*EPHX1* and *ERCC2* polymorphisms are associated with cisplatin induced nephrotoxicity and prognosis in Thai cancer patients” (Manuscript Number: **PONE-D-24-18947**). We appreciate the time and effort you and the reviewers invested in evaluating our work. We have carefully considered the reviewers’ comments and made significant revisions to address their concerns. Below, we outline our responses to each point raised by the reviewers. All corresponding changes that have been added to the manuscript and marked with “Track Changes”.

**Additional Requirements:**

1. *Please ensure that your manuscript meets PLOS ONE's style requirements, including those for file naming. The PLOS ONE style templates can be found at
   https://journals.plos.org/plosone/s/file?id=wjVg/PLOSOne_formatting_sample_main_body.pdf and* [*https://journals.plos.org/plosone/s/file?id=ba62/PLOSOne_formatting_sample_title_authors_affiliations.pdf*](https://journals.plos.org/plosone/s/file?id=ba62/PLOSOne_formatting_sample_title_authors_affiliations.pdf)

**Response to the requirement #1:**

We have revised the formatting according to PLOS ONE’S style requirement.

2. *Thank you for stating the following financial disclosure:*
   *"This research project is supported by Mahidol University, and the National Science Research and Innovation (NSRF) via the Program Management Unit for Human Resources & Institutional Development, Research and Innovation (PMU-B) [Grant number B01F650006]; grant numbers BCG.659 and B05F650041 (NJ)." Please state what role the funders took in the study.  If the funders had no role, please state: "**The funders had no role in study design, data collection and analysis, decision to publish, or preparation of the manuscript."  If this statement is not correct you must amend it as needed. 
   Please include this amended Role of Funder statement in your cover letter; we will change the online submission form on your behalf.*

**Response to the requirement #2:**

Please note that the funders had no role in study design, data collection and analysis, decision to publish, or preparation of the manuscript. This statement has been added in both the cover letter and the main manuscript file under the “Funding” heading on page 2. Please see the added text below.

**Funding**

“This research project is supported by Mahidol University, and the National Science Research and Innovation (NSRF) via the Program Management Unit for Human Resources & Institutional Development, Research and Innovation (PMU-B) [Grant numbers B01F650006, BCG.659 and B05F650041] (NJ). SA received “Master Degree Student Research Assistantship” scholarship from the Faculty of Medicine Ramathibodi hospital, Mahidol University. The funders had no role in study design, data collection and analysis, decision to publish, or preparation of the manuscript.”

3. *PLOS requires an ORCID iD for the corresponding author in Editorial Manager on papers submitted after December 6th, 2016. Please ensure that you have an ORCID iD and that it is validated in Editorial Manager. To do this, go to ‘Update my Information’ (in the upper left-hand corner of the main menu), and click on the Fetch/Validate link next to the ORCID field. This will take you to the ORCID site and allow you to create a new iD or authenticate a pre-existing iD in Editorial Manager.*

**Response to the requirement #3:**

The corresponding author’s ORCID iD has been updated in the Editorial Manager.

4. *We note that you have included the phrase “data not shown” in your manuscript. Unfortunately, this does not meet our data sharing requirements. PLOS does not permit references to inaccessible data. We require that authors provide all relevant data within the paper, Supporting Information files, or in an acceptable, public repository. Please add a citation to support this phrase or upload the data that corresponds with these findings to a stable repository (such as Figshare or Dryad) and provide and URLs, DOIs, or accession numbers that may be used to access these data. Or, if the data are not a core part of the research being presented in your study, we ask that you remove the phrase that refers to these data.*

**Response to the requirement #4:**

We apologize for the overlook of this requirement. Since these data do not form core part of our research, we have revised the information in “Results” section line number 409 and 462 on page 16 and 20, respectively. Please see the revised parts below.

“Upon adjusting the model with other clinical factors such as hypertension, diabetes, heart diseases and cerebrovascular diseases and histological types, also showed TC genotype of *EPHX1* rs1051740 to be significantly associated with AKD in both co-dominant and over-dominant models, although the associations were no longer significant after FDR correction“.

“To analyze clinical and genetic factors, multivariate analysis was then performed using the statistically significant variables and potential AKD-associated clinical factors.”

**Reviewer’s Comments:**

***Reviewer #1:*** *In their manuscript PONE-D-24-18947 entitled “EPHX1 and ERCC2 polymorphisms are associated with cisplatin induced nephrotoxicity and prognosis in Thai cancer patients”, the authors have demonstrated that Single Nucleotide Polymorphisms (SNPs) can be used to identify cancer patients who are susceptible to developing cisplatin-induced nephrotoxicity (CIN). Their results reveal an association between EPHX1 rs1051740 and AKD, and confirms the previously reported associations between ERCC2 SNPs and OS." The manuscript is well written, results are clear and the organization of the results makes it easy to follow. The authors have also addressed both strengths and limitations of the study. The article may be suitable for publication in the PLOS ONE journal.*

**Response to Reviewer #1:**

Thank you so much for taking the time to review our manuscript. We do appreciate your constructive comments.

*Specific comments:*

***Minor remark:*** *can you provide a short list of abbreviations?*

**Response to Reviewer #1:**

List of abbreviations used in our manuscript is provided herein and, in the manuscript on page 3.

- **CIN:** Cisplatin Induced Nephrotoxicity
- **AKI:** Acute Kidney Injury
- **AKD:** Acute Kidney Disease
- **SNP:** Single Nucleotide Polymorphisms
- ***OCT2*:** Organic Cation Transporter 2
- ***SLC22A2*:** Solute Carrier Family 22 member 2
- ***ERCC1***: Excision Repair Cross-Complementing 1
- ***ERCC2***: Excision Repair Cross-Complementing 2
- ***EPHX1:*** Epoxide Hydrolase 1
- ***XPD:*** Xeroderma Pigmentosum group D
- **DME:** Drug Metabolizing Enzyme
- **PFS:** Progression-Free Survival
- **OS:** Overall Survival
- **CTCAE-AKI:** Common Criteria Terminology for Adverse Events - Acute Kidney Injury
- **KDIGO:** Kidney Disease Improving Global Outcomes
- **ICD-10:** 10^th^ Revision of International Classification of Diseases
- **T-Rex:** Thai Reference Exome Database
- **MAF:** Minor Allele Frequency
- **FDR:** False Discovery Rate
- **BH-FDR:** Benjamini-Hochberg False Discovery Rate
- **HWE:** Hardy-Weinberg Equilibrium

***Reviewer #2:*** *The manuscript was well written. Authors presented their findings in a clear and intelligible manner that was easy to follow.*

*Specific comments:*

1. *Authors may consider shortening the length of the discussion section to ease readability.*
2. *A data sharing statement was not included.*

**Response to Reviewer #2:**

Thank you very much for your kind comments and pointing out these points. We have revised the text accordingly as follows.

1. The discussion part has been made more concise where applicable. Please see the revised parts below.

Line 576-580, page 24 Revised: “The use of higher cisplatin doses in the past regimens may be one of the reasons for a higher AKI incidence seen in Patimarattananan et al. The recent improvement of hydration and supplementation regimens may also contribute to lower AKI incidence”.

Line 595 – 599, page 25 Revised: “Similar to our patient’s cohort, male sex, and lower baseline eGFR were among AKD progression factors [35]. Furthermore, a recent meta-analysis identified a higher mortality risk in the AKD group compared to the non-AKD group [34]”.

Line 609 – 615, page 25 Revised: “However, comorbidities including hypertension, diabetes mellitus and age did not show significant association in our study, whereas Patimarattananan et al. suggests that hypertension was significantly associated with AKD development. In line with the same study [1], kidney function after cisplatin treatment did not impact the OS in our study”.

Line 628 – 630, page 26 Deleted: “The extent and outcome of polymorphism’s effect varies, for example, decreased activity of *EPHX1* gene is linked to increased risks of having alcohol dependence, liver cirrhosis and breast cancer”.

Line 635 – 638, page 26 Revised: “Lee et al has shown an association between genetically lower epoxide hydrolase activity and an increased risk of tobacco related cancers among smokers”.

Line 656 – 659, page 27 Deleted: “Interestingly, Khrunin et al. who also found rs1051740 heterozygous genotype to be significantly associated with CIN has proposed that *EPHX1* might not be acting alone to cause CIN. There may be another gene such as EPHX2 which might exert its effect on the functions of *EPHX1,* and hence causing CIN”.

Line 711 – 716, page 29 Deleted: “Moreover, studies have shown that both *ERCC1* and *ERCC2* polymorphisms may influence freedom from loco-regional relapse (ffLRR) and survival outcomes in head & neck and lung cancer patients treated with platinum-based chemotherapies. However, the results from prior studies are inconsistent and does not reach a consensus regarding their clinical use, may be due to sample size and population differences”.

2. Data sharing statement has been included on page 2 of the main manuscript as follows.

**Data sharing statement:**

“All relevant data are within the manuscript and its supporting information files. We have removed all identifying information. The genotyping information and clinical metadata required to replicate the reported study findings can be found in S7 Data file.”

***Reviewer #3:*** *This manuscript presents significant findings on the genetic predictors of cisplatin-induced nephrotoxicity in Thai cancer patients. It is well-structured, ethically compliant, and methodologically rigorous. The results have considerable potential for clinical application. By identifying genetic markers associated with nephrotoxicity, chemotherapy treatments can be personalized, potentially improving the management of adverse effects. However, there are some limitations that need to be addressed.*

*Specific comments:*

*1. Although the study accounts for age and sex in its logistic regression models, other potential confounders such as the details of the chemotherapy regimen, hydration status during treatment, and concurrent medications were not considered. These factors could greatly affect nephrotoxicity outcomes and should be included in the analysis.*

**Response to Reviewer #3:**

Thank you very much for the positive feedback and valuable suggestions. We do agree with the reviewer that other potential confounders, such as chemotherapy regimen details, hydration status, and concurrent medications, could influence nephrotoxicity outcomes. However, given that our study included patients with various tumor types treated with different cisplatin-containing regimens, it was challenging to uniformly account for these variables in our analysis. Additionally, detailed data on these factors were not consistently available for all patients, and the complexity of chemotherapy regimens and premedication protocols made it difficult to incorporate them comprehensively. We have acknowledged these limitations in the revised manuscript under the ‘Discussion’ section (Lines 722-729, page 30). Please see the added paragraph below.

“While this study provides significant insights into the genetic predictors of cisplatin-induced nephrotoxicity, several limitations should be acknowledged. Although we accounted for age and sex in our logistic regression models, other potential confounding factors, such as details of the chemotherapy regimens, pre-chemotherapy hydration status during treatment, and premedication protocols, were not included in the analysis. This is primarily because our study cohort consisted of patients with various tumor types who were treated with different cisplatin-containing regimens, making it challenging to uniformly account for these variables. These factors are known to influence nephrotoxicity outcomes and may have affected our findings.”

*The study's sample is restricted to Thai cancer patients treated at a single hospital. This geographic and ethnic uniformity limits the generalizability of the findings to a wider population. Differences in SNP frequencies among various ethnic groups mean that the results may not be applicable to non-Thai populations.*

*2. A more detailed discussion of the study’s limitations, including potential biases and the constraints of the analyzed SNPs, would offer a more balanced perspective on the findings and their implications.*

**Response to Reviewer #3:**

We appreciate your insightfulness and your suggestions regarding the study’s limitation. We have added the potential limitations and biases in more details in the Discussion (Lines 730 -732, page 30). Please see the revised part below.

“Additionally, the small sample size, single-institution retrospective study design, and limited number of SNPs analyzed, all of which are common in Thais may limit the generalizability of our findings to a wider population.”

Additionally, we have revised the last paragraph of the Discussion (Lines 734-743, page 30) to better summarize our findings and their potential application, as shown below:

“In summary, we demonstrated for the first time that *EPHX1* rs1051740 heterozygous genotype is significantly associated with increased risk of AKD in co-dominant and over-dominant model among Thai cancer patients who received cisplatin. Furthermore, *ERCC2* rs13181 and rs1799793 homozygous major are also confirmed to be associated with better OS in Thai HNSCC patients. Despite certain limitations, these findings highlight potential prognostic value of the analyzed SNPs, helping clinicians preliminarily identify patients more prone to developing AKD during cisplatin-based treatment, particularly in cases of locally advanced HNSCC, where cisplatin remains a mainstay of concurrent chemoradiotherapy (56). Larger prospective studies across diverse populations are necessary to validate these genetic markers for personalized treatment approaches.”

**Reference:**

56. Ngamphaiboon, N., et al., Evolving role of novel radiosensitizers and immune checkpoint inhibitors in (chemo)radiotherapy of locally advanced head and neck squamous cell carcinoma. Oral Oncol, 2023. 145: p. 106520.

We believe that these revisions based on the reviewers’ comments have substantially improved the quality of our manuscript. In addition to addressing the specific comments raised by the reviewers, we have also revised the manuscript according to the journal’s requirements.

We hope that the revised version of our manuscript, along with our detailed responses, can clearly address the comments from the editor and reviewers and are sufficient to make our manuscript suitable for publication in PLOS ONE.

Again, thank you very much for the opportunity to revise and resubmit our manuscript to PLOS ONE. We look forward to receiving your reply.

Sincerely,

Natini Jinawath, MD, PhD

Corresponding author
