# Supplementary material for: EPHX1 and ERCC2 polymorphisms are associated with cisplatin-induced nephrotoxicity and prognosis in Thai cancer patients
Source: PLoS One. 2025 Jun 17;20(6):e0324699. doi: 10.1371/journal.pone.0324699 (PMC12173183; doi:10.1371/journal.pone.0324699)
Supplement: Attachment — Submitted filename: Response_to_Reviewers_auresp_2.docx [file pone.0324699.s015.docx]

Dear the Editor in Chief of PLOS ONE,

Thank you for your kind consideration of our manuscript entitled “*EPHX1* and *ERCC2* polymorphisms are associated with cisplatin induced nephrotoxicity and prognosis in Thai cancer patients” (Manuscript Number: **PONE-D-24-18947R1**). We appreciate the time and effort you and the reviewers invested in evaluating our work. We have carefully considered the reviewers’ comments and made significant revisions to address their concerns. Below, we outline our responses to each point raised by the reviewers. All corresponding changes that have been added to the manuscript and marked with “Track Changes”.

**Reviewer’s Comments:**

***Reviewer #4:***  *The authors report the results of the study “EPHX1 and ERCC2 polymorphisms are associated with cisplatin-induced nephrotoxicity and prognosis in Thai cancer patients”. The authors have evaluated the effects of polymorphisms of six SNPs in the drug-metabolizing enzyme genes, SLC22A2 (rs316019) & EPHX1 (rs1051740), and the DNA repair genes, ERCC1 (rs11615 & rs3212986) & ERCC2 (rs13181 & rs1799793), and CIN in the 169 Thai patients with head and neck, lung, or esophageal cancer and whether they could on predict the cumulative incidence of AKD, progression-free survival (PFS), and overall survival (OS).*

*While acknowledging the research problem's significance and the contextualisation of results within the existing literature, it is essential to address certain ambiguities in the manuscript. The following significant concerns warrant the authors' attention:
A significant area for improvement of the manuscript is regarding the suitability of the study population to reach the study goal. The sample needs to be more important to achieve the primary objectives. Second, the manuscript is written poorly– which complicates the reading and the precise interpretation of the data.*

*I have significant comments for this manuscript:*

*1) The manuscript's language is acceptable but needs to be re-revised; there are word errors on the paper, so it needs to be controlled and corrected.*

**Response to the Reviewer # 4:**

Thank you for your kind suggestion. We have thoroughly checked the grammar and corrected the writing throughout the manuscript, where applicable:

Page 5 – Lines 126-130: “Despite being an effective anticancer drug, its usage can be delayed or terminated because of associated toxicities such as neurotoxicity, gastrointestinal toxicity, ototoxicity and nephrotoxicity [1]. Platinum could be retained in the plasma for as long as 20 years after discontinuation of cisplatin-based chemotherapy [2, 3], thus increasing concerns about the long-term nephrotoxicity risks [4].”

Pages 5-6 – Lines 143-152: “Clinically, CIN manifests in various forms, with Acute Kidney Injury (AKI) being the most common, occurring in 20-30% of patients [7]. Animal models of CIN demonstrates increased serum creatinine (SCr) and blood urea nitrogen, along with histopathological changes [8]. Though SCr is the main marker for renal function evaluation, it needs to be considered in conjunction with other factors such as age and sex. CIN is dose-dependent, with lower doses of cisplatin causing delayed kidney damage as compared to higher doses [9]. Cisplatin dosing regimens vary by cancer type, leading to differences in nephrotoxicity incidence. In lung and esophageal cancer treatments, ≥ 60 mg/m^2^ every 3 - 4 weeks and 75 mg/m^2^ on days 1 and 29 are administered, respectively [10, 11]. Gradually, in HNSCC, weekly 40 mg/m^2^ or 100mg/m^2^ every 3 weeks is being administered concurrently with radiotherapy [12].”

Page 6 – Line 154-159: “AKI may occur within 7 days of cisplatin administration, presented as increase in SCr, whereas Acute Kidney Disease (AKD) develops between 7 and 90 days of cisplatin initiation [13]. Prior studies have commonly used AKI as an endpoint for CIN based on different grading criteria; while the long-term or delayed effects of CIN are not widely studied. AKD, defined as renal damage occurring within 3 months of cisplatin administration, may prove to be a valuable endpoint for studying its delayed nephrotoxic effects [1, 14].”

Page 7 – Lines 178-185: “*ERCC1* and *ERCC2* or *XPD* are integral part of the NER pathway. SNPs in these genes - *ERCC1* rs11615 and rs3212986, and *ERCC2* rs13181 and rs1799793 - are linked to changes in the DNA repair process. Variations in these genes may cause a decrease in the helicase activity and DNA repair. *ERCC1* plays a role in incising the DNA damage site, and can cause a rate-limiting effect, while *ERCC2,* is involved in helicase unwinding the DNA at the damaged site. Hence, these SNPs may reduce the efficiency of DNA repair mechanisms, leading to greater platinum-DNA adduct formation and hindered nephron repair following cisplatin exposure [19].”

Page 25 – Lines 576-580: “We found *EPHX1* rs1051740 significantly associated with cisplatin-induced AKD in co-dominant and over-dominant model, particularly in TC pattern. To our knowledge, no prior studies have performed AKD association analysis with genetic polymorphisms. Multivariate analysis, identified lower baseline eGFR less than 90 ml/min/1.73m^2^ as another factor influencing AKD development after cisplatin treatment.”

Page 25 – Lines 586-596: “No consensus has been reached regarding SNPs associated with CIN, partly due to significant variations in minor allele frequencies across populations. Previous studies have shown association of these 6 selected SNPS with CIN using AKI as the endpoint. Notably, the minor allele frequency of *EPHX1* rs1051740 differs in the Thai population compared to other populations [36]. Most commonly the reference allele is T, whose expression as homozygous TT genotype is associated with faster metabolism than CC. However, in Thai population, T is the minor allele, with a frequency of 0.49. The *EPHX1* rs1051740 (c.337T>C), is one of the 2 widely studied polymorphisms of the *EPHX1* gene. This variant results in a tyrosine-to- histidine substitution at position 113, leading to a 50% reduction in enzyme activity [37]. Decreased enzymatic activity may reduce the detoxification, increasing the formation of highly reactive metabolites, making the cells more susceptible to challenges.”

Page 26 – Lines 603-607: “Another meta-analysis found an association between rs1051740 polymorphism and HNSCC risk in population-based studies. [18]. While *EPHX1* is well-studied regarding cancer risk, few studies have explored its relationship with CIN. To date, only Khrunin et al. have reported a higher frequency of CIN in ovarian cancer patients with the rs1051740 heterozygous genotype in a Russian population [15].”

Pages 26-27 – Lines 621-640: “*ERCC1* and *ERCC2* are key components of the NER pathway, activated by the formation of DNA adducts. Genetic variations in these genes may influence DNA repair capacity. Synonymous variants in the 3’ UTR region such as *ERCC1* rs11615 and rs3212986, can affect protein stability, folding, structure, and expression [19, 45]. Previous studies by Khrunin et al. and Tzvetkov et al. reported an increased risk of CIN in ovarian and other cancers related to *ERCC1* rs11615 and rs3212986 in Caucasian [25, 26]. However, Zazuli et al. found *ERCC1* rs3212986 to reduce risk of CIN in Caucasian testicular cancer patients [17]. In East-Asians, Chen et al. found no significant association between CIN and *ERCC1* rs11615 in Chinese lung cancer patients [46]. The association of *ERCC2* with CIN has also been widely studied, but results vary due to differing endpoints and populations. Studies have shown an increased risk of CIN in Caucasian osteosarcoma and gastric cancer patients with *ERCC2* rs13181 and rs1799793, respectively [27, 47], while in East-Asian lung cancer patients, Kim et al. did not find any association between CIN and *ERCC2* rs13181 [48]. *SLC22A2* rs316019, a nonsynonymous missense variant (p.270Ala>Ser; c.808G>T), impacts cisplatin uptake in renal proximal tubules and has been linked to CIN in multiple studies [16, 17, 21-24]. However, the reported effects of *SLC22A2* rs316019 on CIN vary across populations and outcome definitions. Among East-Asians, Zazuli et al. suggested a protective effect of this SNP when using CTCAE-AKI criteria [17]. Notably, all these studies used AKI as the endpoint for CIN. In our study, none of the candidate SNPs from *SLC22A2*, *ERCC1* and *ERCC2* showed a significant association with AKD in any genetic model.”

Pages 27-28 – Lines 642-657: “Since AKD by itself can lead to poor OS in cancer patients, partly due to the resulting inadequate treatment, we further investigated the effect of these candidate SNPs on OS. In our study, although the OS analysis of AKD vs non-AKD groups was not statistically significant, we observed a trend indicating that patients without AKD had numerically longer OS compared to those with AKD. OS analysis for each selected SNP revealed no association with polymorphisms in *SLC22A2*, *EPHX1* and *ERCC1* genes. However, using a dominant genetic model, OS was associated with *ERCC2* rs13181 and rs1799793: patients with *ERCC2* rs13181-TT genotype and rs1799793-CC genotype exhibited better OS. Notably, these *ERCC2* SNPs showed no association with AKD, suggesting their effects on OS are independent of AKD status. Previously, data on survival in relation to *ERCC2* gene polymorphisms have varied across different populations and cancer types, limiting their application in clinical practice. Variant genotypes of *ERCC2* rs13181 and rs1799793 have been significantly associated with poor survival in Chinese NSCLC patients [49]. A meta-analysis by Yang et al. found that the rs13181 variant G and rs1799793 variant T were poorly associated with OS in NSCLC patients [50]. Among Caucasian gastric cancer patients, rs13181 was not associated with OS, whereas the rs1799793 homozygous variant AA genotype was significantly linked to poor OS [51].”

Page 28 – Lines 659-670: “Stratification of OS analysis by cancer type revealed that both SNPs of *ERCC2* were associated only with HNSCC patients in our cohort under a dominant genetic model. Our findings align with recent studies reporting worse OS for HNSCC patients with the *ERCC2* rs13181 homozygous minor genotype and significantly better OS for those with the rs1799793 homozygous major genotype under the same model [52, 53]. OS stratification for lung cancer patients showed no significant associations in our study. However, an Asian lung cancer study recently reported that *ERCC2* rs13181 TG and TG+GG genotypes were significantly linked to worse OS under a dominant model [54]. These differing results may stem from the small sample size in our lung cancer cohort. While our findings showed no OS association with *ERCC1* rs11615 and rs3212986, these SNPs have been widely studied in various cancers with varying survival outcomes. The G allele of rs11615has been significantly associated with better OS, and the A allele of rs3212986 has been linked to improved PFS in a lung cancer study [55].”

***Reviewer#4:***

*2) What was the enrollment period? Were all consecutive patients enrolled, and when were the data and follow-up censored?*

**Response to the Reviewer # 4:**

Thank you for giving us the opportunity to elaborate the enrollment period further, please see the revised parts in the manuscript and as follows:

Pages 8-9 – Lines 218-223: “For HNSCC cohort, in our prospective multidisciplinary observation study of HNSCC and NPC patients at Ramathibodi Hospital, Mahidol University, we have accrued eligible patients since 2016. The study enrolled patients with LA-HNSCC and NPC treated according to the standard of care at their treating physician’s discretion. Enrollment was consistent, except during Covid-19 pandemic when the enrollment was hold, and subsequently resumed in 2021. Similarly, lung and esophageal cancer cohorts were also enrolled since 2016.”

Page 11 – Lines 300-301: “The survival status was cross-checked with the National Security Death Index of Thailand on 23/03/2022.”

***Reviewer#4:***

*3) Describe the setting, locations, and relevant dates, including periods of recruitment, exposure, follow-up, and data collection*

**Response to the Reviewer # 4:**

We appreciate the reviewer for their insightful comment. All enrolled patients were treated with cisplatin at Faculty of Medicine, Ramathibodi Hospital, Mahidol University, Bangkok, Thailand and were retrospectively identified with their available blood samples in Ramathibodi Comprehensive Tumor Biobank collected between 1^st^ January 2016 to 30^th^ June 2020. We also described the details in the “Patient Selection” section on Pages 8-9 – Lines 200-223 and here as follows:

“Clinical and treatment data were obtained from an in-house observational head and neck cancer cohort database, Ramathibodi Tumor Registry, pharmacy database, and medical records included patient demographics, serum creatinine (SCr), estimated glomerular filtration rate (eGFR; CKD-EPI), cancer staging and comorbidities (ICD-10). All clinical information was verified by two independent medical oncologists. The clinical data were initially accessed on 15^th^ July 2020. The authors had access to information that could identify individual participants during data collection; however, no individually identifiable data was reported in this study.

Solid tumor patients treated with cisplatin chemotherapy at the Faculty of Medicine, Ramathibodi Hospital, Mahidol University, Bangkok, Thailand were retrospectively identified. Criteria used for patient inclusion were: head & neck, lung and esophageal cancer patients, age ≥18 years, availability of blood samples in Ramathibodi Comprehensive Tumor Biobank collected between 1^st^ January 2016 to 30^th^ June 2020. Samples were collected from the biobank for research purposes starting from 17^th^ March 2021. Patients with known chronic kidney disease as defined by The Kidney Disease Improving Global Outcomes (KDIGO) guideline before the start of cisplatin-based treatment were excluded [14].

For HNSCC cohort, in our prospective multidisciplinary observation study of HNSCC and NPC patients at Ramathibodi Hospital, Mahidol University, we have accrued eligible patients since 2016. The study enrolled patients with LA-HNSCC and NPC treated according to the standard of care at their treating physician’s discretion. Enrollment was consistent, except during Covid-19 pandemic when the enrollment was hold, and subsequently resumed in 2021. Similarly, lung and esophageal cancer cohorts were also enrolled since 2016. The study enrolled patients with LA-HNSCC and NPC treated according to the standard of care at their treating physician’s discretion. Enrollment was consistent, except during Covid-19 pandemic when the enrollment was hold, and subsequently resumed in 2021. Similarly, lung and esophageal cancer cohorts were also enrolled since 2016.”

***Reviewer#4:***

*4) How were the sample size and power calculated for this study?*

**Response to the Reviewer # 4:**

Thank you for your comment. In our study, sample size and power were calculated using the STATA program, assuming 25% nephrotoxicity, which indicated 80% power in 169 patients. This allowed us to identify a statistically significant association between selected SNPs and AKD.

We have also mentioned power and sample size calculation on Page 11 – Lines 282 - 284 and here as follows:

“Power calculation showed that 169 patients assuming 25% nephrotoxicity would give our study 80% power to identify statistically significant association between selected SNPs and AKD with an alpha of 0.05 and odds ratio of 2.8.”

***Reviewer#4:***

*5) A CONSORT diagram would help better understand which patients were removed from analyses for various reasons.*

**Response to the Reviewer # 4:**

Thank you for your kind suggestion, we have added a CONSORT diagram as Figure 1 in the main figures and as follows:

***Reviewer#4:***

*6) What were the patients' side effects after receiving the platinum chemotherapy? Please elaborate*

**Response to the Reviewer # 4:**

We sincerely thank the reviewer for their insightful comment regarding the side effects experienced by patients after receiving platinum chemotherapy. In this study, we specifically focused on SNPs associated with cisplatin-induced nephrotoxicity. Consequently, the only toxicity data collected and analyzed pertained to nephrotoxicity. Unfortunately, information on other toxicities was not available in this dataset.

We acknowledge the importance of capturing a broader spectrum of chemotherapy-related toxicities and will consider this aspect for future research to provide a more comprehensive analysis.

***Reviewer#4:***

*7) Since chemotherapy is associated with drug toxicity, how dose delay or discontinuance was accounted for?*

**Response to the Reviewer # 4:**

Thank you for your valuable comment. Consistently accounting for this potential confounder was challenging due to the varying dosing regimens across tumor types. We acknowledge it’s impact on the findings and will incorporate it into future analyses for enhanced thoroughness.

***Reviewer#4:***

*8) The authors have calculated the odds ratio to calculate the risk. Did the authors adjust the odds with other clinical covariates? Adjusting the odds with different covariates impacts clinical parameters and is crucial.*

**Response to the Reviewer # 4:**

We appreciate your comment and agree that adjusting the odds ratio with different covariates is crucial. In our analysis, the odds ratio was adjusted for other clinical covariates; however, the findings were similar to those obtained when adjusting for age and sex. We have elaborated the adjusted clinical covariates in the manuscript on Page 16 – Lines 415 – 418 and as follows:

“Upon adjusting the model with other clinical factors such as hypertension, diabetes, heart diseases and cerebrovascular diseases and histological types, also showed TC genotype of *EPHX1* rs1051740 to be significantly associated with AKD in both co-dominant and over-dominant models, although the associations were no longer significant after FDR correction.”

***Reviewer#4:***

*9) The therapeutic response and survival depend on tumour grade and stage; how was this factor accounted for in the data analysis*

**Response to the Reviewer # 4:**

Thank you for raising this concern. We do agree that survival depends on tumor grade and stage. Tumor grade was not always reported by the pathologists in our institute, and hence, there was some missing data. However, we have included staging in our analyses.

***Reviewer#4:***

*10) Cox regression analysis results should be shown (in tables, at least as supplementary data)*

**Response to the Reviewer # 4:**

Thank you for your invaluable suggestion. We performed Cox regression analysis for overall survival and time-to-AKD. The results have been added in the “Results” section on Page 20-21 – Lines: 460-466, Page 22-23 – Lines: 519-527 and tables in the supplementary data as Supplementary Table 7 and 8 and as follows respectively:

Page 20-21 – Lines: 460-466: “In addition, multivariate Cox regression analysis using significant variables were performed and identified age, baseline eGFR, SCr and individuals carrying TC genotypes of the *EPHX1* rs1051740 SNP as significant in univariate analysis [HR 1.040, CI 95% 1.006 – 1.076, *P* = 0.019; HR 0.954, CI 95% 0.934 – 0.974, *P* = <0.001; HR 14.490, CI 95% 2.167 – 96.873, *P* = 0.006; HR 2.433, 1.012 – 5.847, *P* = 0.047, respectively]. However, none of the variables analyzed were identified as independent factors of time-to-AKD after multivariate Cox analysis. The associated risk factors of time-to-AKD are listed in S7 Table.”

Page 22-23 – Lines: 519-527: “Multivariate Cox regression was also performed to analyze the significant variables and potential risk factors for OS, and identified male , diabetes mellitus, heart disease, G allele on *ERCC2* rs13181 and T allele on rs1799793 tended to have an increased risk of mortality [HR 1.815, 95% CI 1.077-3.058, *P* = 0.025; HR 2.219, 95% CI 1.133 – 4.343, *P* = 0.020; HR 2.674, CI 95% 1.222 – 5.851, *P* = 0.014; HR 2.080, CI 95% 1.270 – 3.407, *P* = 0.004; HR 2.107, CI 95% 1.238 – 3.586, *P* = 0.006, respectively] whereas NSCC had decreased mortality risk [HR 0.508, CI 95% 0.279 – 0.925, *P* = 0.027] in the univariate Cox regression model, but this did not retain statistical significance in the multivariate Cox proportional hazards model. List of associated factors affecting mortality are given in S8 Table.”

**Supplementary Table 7: Associated Risk Factors of Time-To-AKD in this cohort.**

| **Factors** | **AKD (n=43)**  **(25.4%)** | **Non-AKD**  **(n=126) (74.6%)** | **Univariate Cox regression HR**  **(95% CI), *P* value** | **Multivariate Cox regression HR**  **(95% CI), *P* value** |
| --- | --- | --- | --- | --- |
| Male | 25 (58.1) | 85 (67.5) | 0.711 (0.387 – 1.303), 0.270 | **-** |
| **Age**  **Age group**  <65 | -  34 (79.1) | -  105 (83.3) | **1.040 (1.006 – 1.076), 0.019**  0.752 (0.361 – 1.570), 0.449 | 0.995 (0.950 – 1.042), 0.855  - |
| **Comorbidity (ICD-10)**  Hypertension  Diabetes Mellitus  Cerebrovascular Disease  Heart Disease | 17 (39.5)  4 (9.3)  4 (9.3)  2 (4.7) | 33 (26.2)  11 (8.7)  9 (7.1)  7 (5.6) | 1.630 (0.884 – 3.005), 0.117  0.992 (0.354 – 2.779), 0.989  1.330 (0.474 – 3.729), 0.588  0.828 (0.200 – 3.426), 0.795 | -  -  -  - |
| **Histology**  Non-SCC | 14 (32.6) | 31 (24.6) | 1.394 (0.736 – 2.639), 0.307 | - |
| **Staging Group**  3 – 4 | 34 (25.4) | 100 (74.6) | 0.942 (0.451 – 1.964), 0.874 | - |
| **Baseline mean eGFR (ml/min/1.73m^2^ )** | - | - | **0.954 (0.934 – 0.974), <0.001** | **0.947 (0.910 – 0.985), 0.008** |
| **Baseline mean SCr (mg/dL)** | - | - | **14.490 (2.167 – 96.873), 0.006** | 0.368 (0.174 – 7.796), 0.522 |
| **rs316019**  **AC**  **AA** | 11 (25.6)  0 | 22 (17.5)  3 (2.4) | 1.450 (0.730 – 2.878), 0.288  - | -  - |
| **rs1051740**  **TC**  **TT** | 30 (69.8)  7 (16.2) | 57 (45.2)  36 (28.6) | **2.433 (1.012 – 5.847), 0.047**  0.997 (0.335 – 2.971), 0.997 | 2.289 (0.947 – 5.535), 0.066  0.988 (0.331 – 2.949), 0.984 |
| **rs11615**  **AG**  **AA** | 17 (39.5)  5 (11.6) | 57 (45.2)  10 (8.0) | 0.892 (0.470 – 1.692), 0.727  1.365 (0.514 – 3.624), 0.531 | -  - |
| **rs3212986**  **CA**  **AA** | 17 (39.5)  6 (14.0) | 49 (38.8)  15 (12.0) | 1.077 (0.564 – 2.057), 0.822  1.245 (0.499 – 3.105), 0.638 | -  - |
| **rs13181**  **TG**  **GG** | 6 (14.0)  0 | 29 (23.0)  2 (1.6) | 0.558 (0.235 – 1.323), 0.186  **-** | -  - |
| **rs1799793**  **CT**  **TT** | 3 (7.0)  1 (2.3) | 22 (17.5)  1 (0.8) | 0.374 (0.115 – 1.212), 0.101  3.820 (0.514 – 28.372), 0.190 | -  - |

**Supplementary Table 8: Risk Factors of Overall Survival**

| **Factors** | **Alive (n=94)**  **(55.6%)** | **Death**  **(n=75) (44.4%)** | **Univariate Cox regression HR**  **(95% CI), *P* value** | **Multivariate Cox regression HR**  **(95% CI), *P* value** |
| --- | --- | --- | --- | --- |
| Male | 54 (49.1) | 56 (50.9) | **1.815 (1.077 – 3.058), 0.025** | 1.455 (0.822 – 2.574), 0.197 |
| **Median Age (range)**  **Age group**  <65 | -  76 (54.7) | -  63 (45.3) | 1.021 (0.996 – 1.046), 0.088  0.933 (0.503 – 1.732), 0.828 | -  - |
| **Comorbidity (ICD-10)**  Hypertension  Diabetes Mellitus  Cerebrovascular Disease  Heart Disease | 29 (58.0)  5 (33.3)  7 (53.9)  2 (22.2) | 21 (42.0)  10 (66.7)  6 (46.2)  7 (77.8) | 0.912 (0.551 – 1.511), 0.723  **2.219 (1.133 – 4.343), 0.020**  1.289 (0.559 – 2.970), 0.551  **2.674 (1.222 – 5.851), 0.014** | ­-  1.687 (0.837 – 3.401), 0.143  -  1.780 (0.788 – 4.023), 0.165 |
| **Histology**  Non-SCC | 32 (71.1) | 13 (28.9) | **0.508 (0.279 – 0.925), 0.027** | 0.649 (0.339 – 1.242), 0.192 |
| **Staging Group**  3 – 4 | 69 (51.5) | 65 (48.5) | 1.882 (0.967 – 3.665), 0.063 | - |
| **Baseline mean eGFR (ml/min/1.73m^2^ )** | - | - | 0.998 (0.982 – 1.014), 0.849 | **-** |
| **Baseline mean SCr (mg/dL)** | - | - | 1.281 (0.314 – 5.213), 0.729 | **-** |
| **rs316019 A Carriage** | 18 (50.0) | 18 (50.0) | 1.386 (0.815 – 2.356), 0.228 | - |
| **rs1051740 T Carriage** | 73 (56.2) | 57 (43.8) | 0.883 (0.520 – 1.502), 0.649 | - |
| **rs11615 A Carriage** | 53 (59.6) | 36 (40.4) | 0.845 (0.537 – 1.330), 0.468 | - |
| **rs3212986 A Carriage** | 46 (52.9) | 41 (47.1) | 1.117 (0.708 – 1.760), 0.633 | - |
| **rs13181 G Carriage** | 14 (37.8) | 23 (62.2) | **2.080 (1.270 – 3.407), 0.004** | 1.837 (0.918 – 3.674), 0.086 |
| **rs1799793 T Carriage** | 9 (33.3) | 18 (66.7) | **2.107 (1.238 – 3.586), 0.006** | 1.029 (0.477 – 2.220), 0.940 |

***Reviewer#4:***

*11) Another validation cohort should be used to validate the discovery cohort.*

**Response to the Reviewer # 4:**

These SNPs are known to be associated with AKI in other populations and other cancer types. We showed that these SNPs were also associated with AKD in HNSCC, Lung, and ESCC for the first time in Thais. We do agree that the findings should be validated in a larger, independent patient cohort before it can be implemented in clinics. We are now collecting clinical information prospectively from a continuing observatory cohort of HNSCC patients to validate our current findings.

***Reviewer#4:***

*12) Insert in the conclusion a sentence or a short sentence that summarises the relevance of the results found in this study. It is essential to emphasise the significance of the results obtained from the objectives proposed by the authors.*

**Response to the Reviewer # 4:**

We appreciate your comment to make summary clearer. We have revised relevant parts to emphasize the significance on Page 29 – Lines 695-698 and added a key findings table on Page 30 and as follows respectively:

“The AKD-associated SNPs can be utilized to tailor treatment protocols for patients undergoing cisplatin-based therapy, advancing personalized chemotherapy approaches Table 5.”

**Table 5. Summary of key findings and their clinical implications**

| **Key Findings** | **Potential clinical implications** |
| --- | --- |
| ***EPHX1***  rs1051740 | A tailored treatment protocol to reduce potential AKD occurrence (personalized chemotherapy approach) |
| ***ERCC2***  rs13181  rs1799793 | Prognostic biomarkers for HNSCC patients |

***Reviewer#4:***

*13) No attempts have been made to collect information on the other confounding factors which would have influenced for genotyping variants.*

**Response to the Reviewer # 4:**

Thank you for your comment. We agree with the reviewer that other confounding factors such as chemotherapy regimens, hydration status, co-medications or co-morbidities might have influenced. Our study cohort consisted of patients with various tumor types and different cisplatin-containing regimens, hence these variables were difficult to be accounted for uniformly. We have also added this as a limitation in the manuscript on Page 29 – Lines 675-681 and here as follows:

“Although we accounted for age and sex in our logistic regression models, other potential confounding factors, such as details of the chemotherapy regimens, pre-chemotherapy hydration status during treatment, and premedication protocols, were not included in the analysis. This is primarily because our study cohort consisted of patients with various tumor types who were treated with different cisplatin-containing regimens, making it challenging to uniformly account for these variables. These factors are known to influence nephrotoxicity outcomes and may have affected our findings.”

***Reviewer#4:***

*14) Some limitations of the study should be discussed*

**Response to the Reviewer # 4:**

Thank you for your comment. We have added the study limitations in the “Discussion” part of our manuscript on Page 28-29 – Lines 674-685 and here as follows:

“While this study provides significant insights into the genetic predictors of cisplatin-induced nephrotoxicity, several limitations should be acknowledged. Although we accounted for age and sex in our logistic regression models, other potential confounding factors, such as details of the chemotherapy regimens, pre-chemotherapy hydration status during treatment, and premedication protocols, were not included in the analysis. This is primarily because our study cohort consisted of patients with various tumor types who were treated with different cisplatin-containing regimens, making it challenging to uniformly account for these variables. These factors are known to influence nephrotoxicity outcomes and may have affected our findings. Additionally, the small sample size, single-institution retrospective study design, and limited number of SNPs, all of which are common in Thais, may restrict the generalizability of our findings to broader populations, highlighting the need for validation in larger and more diverse cohorts.”

***Reviewer #5:*** *General Comments:*

*The manuscript, titled "EPHX1 and ERCC2 polymorphisms are associated with cisplatin-induced nephrotoxicity and prognosis in Thai cancer patients", explores an important clinical issue regarding genetic predisposition to cisplatin-induced nephrotoxicity. The study is well-structured and provides significant findings, particularly concerning the association of EPHX1 rs1051740 and ERCC2 SNPs with nephrotoxicity and survival outcomes.

The following comments address the review questions and include additional observations:

Specific Comments on the Manuscript:*

***Reviewer #5:***

*Clarity of Hypothesis and Objectives:
The manuscript effectively outlines its objectives and hypotheses, focusing on SNPs linked to nephrotoxicity in Thai cancer patients. However, a clearer differentiation between novel findings and confirmation of existing knowledge would enhance the manuscript's impact.*

**Response to the Reviewer # 5:**

Thank you for your kind comment and suggestion to clarify the findings for better understanding. We have revised the relevant parts for easier understanding on Page 29 – Lines 687-698 and as follows:

“In summary, we demonstrated for the first time that *EPHX1* rs1051740 heterozygous genotype is significantly associated with increased risk of AKD in co-dominant and over-dominant model among Thai cancer patients who received cisplatin. Furthermore, *ERCC2* rs13181 and rs1799793 homozygous major are also confirmed to be associated with better OS in Thai HNSCC patients Table 5. Despite certain limitations, these findings highlight potential prognostic value of the analyzed SNPs, helping clinicians preliminarily identify patients more prone to developing AKD during cisplatin-based treatment, particularly in cases of locally advanced HNSCC, where cisplatin remains a mainstay of concurrent chemoradiotherapy [56]. The AKD-associated SNPs can be utilized to tailor treatment protocols for patients undergoing cisplatin-based therapy, advancing personalized chemotherapy approaches Table 5. Larger prospective studies across diverse populations are necessary to validate these genetic markers for personalized treatment approaches.”

***Reviewer #5:***

Data Availability and Sharing:
While the authors have addressed data sharing requirements, ensuring that the data and supplementary files (e.g., S7 Data file) are easily accessible is crucial. Clarification regarding the repository details would improve transparency.

**Response to the Reviewer # 5:**

Thank you for bringing our attention to data availability and sharing. All raw data required for reproducing the results are in S9 Data file (previously S7 Data file), which can be downloaded from the supplement file download link when the manuscript is published.

***Reviewer #5:***

Methods:
A) The methods are detailed and appropriate for the study's aims. However, the choice of SNPs and their allele frequencies should be briefly discussed in the context of Thai population genetics.

**Response to the Reviewer # 5:**

We sincerely appreciate your comment. SNPs analyzed in prior studies for cisplatin-induced nephrotoxicity were selected. Further based on minor allele frequency (MAF) ≥ 5% in Thai population (obtained from Thai Reference Exome Database (T-REx), candidate SNPs were selected and analyzed. We have also elaborated the choice of SNPs in the “SNP Selection” part in the manuscript on Pages 9-10 – Lines 245-252 and Table 2 on Page 16 and as follows respectively:

Pages 9-10 – Lines 245-252: “Previous clinical studies and systematic reviews related to CIN-associated SNPs in different types of cancers and populations in PubMed from 2008 – 2020 were reviewed [4, 15-17, 21-29]. Only the candidate SNPs with minor allele frequency (MAF) ≥ 5% in Thai population were included. Finally, a total of 6 SNPs in 4 genes were selected. Allele frequencies in Thai population were obtained from the Thai Reference Exome Database (T-REx), containing 1,092 Thai individuals (accessed on 25^th^ February 2022). *SLC22A2* rs316019 and *EPHX1* rs1051740 are involved in drug metabolism, while *ERCC1* rs11615 and rs3212986, and *ERCC2* rs13181 and rs1799793 play an important part in DNA repair mechanisms.”

**Table 2. Information and allele frequencies of the 6 selected SNPs in Thai population.** Allele frequencies were calculated based on the T-REx database [30].

| **Gene** | **SNP** | **SNP Information** | **T-REx Frequencies** | | **Data Allele Frequencies** | | **HWE *P* value** |
| --- | --- | --- | --- | --- | --- | --- | --- |
| ***SLC22A2*** | **rs316019** | Missense c.808G>T (p.270Ala > Ser) | C | A | C | A | 0.187 |
|  |  |  | 0.890 | 0.110 | 0.8289 | 0.1711 |  |
| ***EPXH1*** | **rs1051740** | Missense c.337T>C (p.Tyr113His) | C | T | C | T | 0.331 |
|  |  |  | 0.504 | 0.496 | 0.5036 | 0.4964 |  |
| ***ERCC1*** | **rs11615** | Synonymous c.354T>C (p.Asn118Asn) | G | A | G | A | 0.482 |
|  |  |  | 0.697 | 0.303 | 0.6000 | 0.4000 |  |
| ***ERCC1*** | **rs3212986** | Synonymous 3’ UTR c*197G>T | C | A | C | A | 0.261 |
|  |  |  | 0.664 | 0.336 | 0.7205 | 0.2795 |  |
| ***ERCC2*** | **rs13181** | Missense c.2251A>C (p.751lys > Gln) | T | G | T | G | 0.910 |
|  |  |  | 0.901 | 0.099 | 0.9108 | 0.0892 |  |
| ***ERCC2*** | **rs1799793** | Missense c.934G>A (p.Asp312Asn) | C | T | C | T | 0.889 |
|  |  |  | 0.937 | 0.063 | 0.9422 | 0.0578 |  |

HWE *P*, Hardy-Weinberg Equilibrium *P* value of the non-AKD (control) group.

***Reviewer #5:***

*B) Missing details on hydration regimens, concurrent medications, and cisplatin dosing specifics should be acknowledged as potential confounders.*

**Response to the Reviewer # 5:**

Thank you for your valuable comment. We do agree that hydration regimens, concurrent medications, and cisplatin dosing are potential confounders. This indeed is a limitation in our study and have discussed these in the manuscript on Page 28-29 – Lines 675-681 and here as follows:

“Although we accounted for age and sex in our logistic regression models, other potential confounding factors, such as details of the chemotherapy regimens, pre-chemotherapy hydration status during treatment, and premedication protocols, were not included in the analysis. This is primarily because our study cohort consisted of patients with various tumor types who were treated with different cisplatin-containing regimens, making it challenging to uniformly account for these variables. These factors are known to influence nephrotoxicity outcomes and may have affected our findings.”

***Reviewer #5:***

Results:
A) The results are systematically presented, but some sections could benefit from additional context:
Highlight the clinical relevance of the significant findings, especially the EPHX1 rs1051740 association with AKD.

**Response to the Reviewer # 5:**

Thank you for your kind suggestion. We have revised relevant parts to emphasize the significance of our findings on Page 29 – Lines 695-698 and added a key findings table on Page 30 and as follows respectively:

“The AKD-associated SNPs can be utilized to tailor treatment protocols for patients undergoing cisplatin-based therapy, advancing personalized chemotherapy approaches Table 5.”

**Table 5. Summary of key findings and their clinical implications**

| **Key Findings** | **Potential clinical implications** |
| --- | --- |
| ***EPHX1***  rs1051740 | A tailored treatment protocol to reduce potential AKD occurrence (personalized chemotherapy approach) |
| ***ERCC2***  rs13181  rs1799793 | Prognostic biomarkers for HNSCC patients |

***Reviewer #5:***

*The Kaplan-Meier curves and survival analyses are insightful, though more detailed figures or supplementary visuals might aid interpretation.*

**Response to the Reviewer # 5:**

We sincerely appreciate your comment. We have additionally performed Cox analyses that may aid in better interpretation of our survival analyses. The results have been added in the “Results” section on Page 20-21 – Lines: 460-466, Page 22-23 – Lines: 519-527 and tables in the supplementary data as Supplementary Table 7 and 8 and as follows respectively:

Page 20-21 – Lines: 460-466: “In addition, multivariate Cox regression analysis using significant variables were performed and identified age, baseline eGFR, SCr and individuals carrying TC genotypes of the *EPHX1* rs1051740 SNP as significant in univariate analysis [HR 1.040, CI 95% 1.006 – 1.076, *P* = 0.019; HR 0.954, CI 95% 0.934 – 0.974, *P* = <0.001; HR 14.490, CI 95% 2.167 – 96.873, *P* = 0.006; HR 2.433, 1.012 – 5.847, *P* = 0.047, respectively]. However, none of the variables analyzed were identified as independent factors of time-to-AKD after multivariate Cox analysis. The associated risk factors of time-to-AKD are listed in S7 Table.”

Page 22-23 – Lines: 519-527: “Multivariate Cox regression was also performed to analyze the significant variables and potential risk factors for OS, and identified male , diabetes mellitus, heart disease, G allele on *ERCC2* rs13181 and T allele on rs1799793 tended to have an increased risk of mortality [HR 1.815, 95% CI 1.077-3.058, *P* = 0.025; HR 2.219, 95% CI 1.133 – 4.343, *P* = 0.020; HR 2.674, CI 95% 1.222 – 5.851, *P* = 0.014; HR 2.080, CI 95% 1.270 – 3.407, *P* = 0.004; HR 2.107, CI 95% 1.238 – 3.586, *P* = 0.006, respectively] whereas NSCC had decreased mortality risk [HR 0.508, CI 95% 0.279 – 0.925, *P* = 0.027] in the univariate Cox regression model, but this did not retain statistical significance in the multivariate Cox proportional hazards model. List of associated factors affecting mortality are given in S8 Table.”

**Supplementary Table 7: Associated Risk Factors of Time-To-AKD in this cohort.**

| **Factors** | **AKD (n=43)**  **(25.4%)** | **Non-AKD**  **(n=126) (74.6%)** | **Univariate Cox regression HR**  **(95% CI), *P* value** | **Multivariate Cox regression HR**  **(95% CI), *P* value** |
| --- | --- | --- | --- | --- |
| Male | 25 (58.1) | 85 (67.5) | 0.711 (0.387 – 1.303), 0.270 | **-** |
| **Age**  **Age group**  <65 | -  34 (79.1) | -  105 (83.3) | **1.040 (1.006 – 1.076), 0.019**  0.752 (0.361 – 1.570), 0.449 | 0.995 (0.950 – 1.042), 0.855  - |
| **Comorbidity (ICD-10)**  Hypertension  Diabetes Mellitus  Cerebrovascular Disease  Heart Disease | 17 (39.5)  4 (9.3)  4 (9.3)  2 (4.7) | 33 (26.2)  11 (8.7)  9 (7.1)  7 (5.6) | 1.630 (0.884 – 3.005), 0.117  0.992 (0.354 – 2.779), 0.989  1.330 (0.474 – 3.729), 0.588  0.828 (0.200 – 3.426), 0.795 | -  -  -  - |
| **Histology**  Non-SCC | 14 (32.6) | 31 (24.6) | 1.394 (0.736 – 2.639), 0.307 | - |
| **Staging Group**  3 – 4 | 34 (25.4) | 100 (74.6) | 0.942 (0.451 – 1.964), 0.874 | - |
| **Baseline mean eGFR (ml/min/1.73m^2^ )** | - | - | **0.954 (0.934 – 0.974), <0.001** | **0.947 (0.910 – 0.985), 0.008** |
| **Baseline mean SCr (mg/dL)** | - | - | **14.490 (2.167 – 96.873), 0.006** | 0.368 (0.174 – 7.796), 0.522 |
| **rs316019**  **AC**  **AA** | 11 (25.6)  0 | 22 (17.5)  3 (2.4) | 1.450 (0.730 – 2.878), 0.288  - | -  - |
| **rs1051740**  **TC**  **TT** | 30 (69.8)  7 (16.2) | 57 (45.2)  36 (28.6) | **2.433 (1.012 – 5.847), 0.047**  0.997 (0.335 – 2.971), 0.997 | 2.289 (0.947 – 5.535), 0.066  0.988 (0.331 – 2.949), 0.984 |
| **rs11615**  **AG**  **AA** | 17 (39.5)  5 (11.6) | 57 (45.2)  10 (8.0) | 0.892 (0.470 – 1.692), 0.727  1.365 (0.514 – 3.624), 0.531 | -  - |
| **rs3212986**  **CA**  **AA** | 17 (39.5)  6 (14.0) | 49 (38.8)  15 (12.0) | 1.077 (0.564 – 2.057), 0.822  1.245 (0.499 – 3.105), 0.638 | -  - |
| **rs13181**  **TG**  **GG** | 6 (14.0)  0 | 29 (23.0)  2 (1.6) | 0.558 (0.235 – 1.323), 0.186  **-** | -  - |
| **rs1799793**  **CT**  **TT** | 3 (7.0)  1 (2.3) | 22 (17.5)  1 (0.8) | 0.374 (0.115 – 1.212), 0.101  3.820 (0.514 – 28.372), 0.190 | -  - |

**Supplementary Table 8: Risk Factors of Overall Survival**

| **Factors** | **Alive (n=94)**  **(55.6%)** | **Death**  **(n=75) (44.4%)** | **Univariate Cox regression HR**  **(95% CI), *P* value** | **Multivariate Cox regression HR**  **(95% CI), *P* value** |
| --- | --- | --- | --- | --- |
| Male | 54 (49.1) | 56 (50.9) | **1.815 (1.077 – 3.058), 0.025** | 1.455 (0.822 – 2.574), 0.197 |
| **Median Age (range)**  **Age group**  <65 | -  76 (54.7) | -  63 (45.3) | 1.021 (0.996 – 1.046), 0.088  0.933 (0.503 – 1.732), 0.828 | -  - |
| **Comorbidity (ICD-10)**  Hypertension  Diabetes Mellitus  Cerebrovascular Disease  Heart Disease | 29 (58.0)  5 (33.3)  7 (53.9)  2 (22.2) | 21 (42.0)  10 (66.7)  6 (46.2)  7 (77.8) | 0.912 (0.551 – 1.511), 0.723  **2.219 (1.133 – 4.343), 0.020**  1.289 (0.559 – 2.970), 0.551  **2.674 (1.222 – 5.851), 0.014** | ­-  1.687 (0.837 – 3.401), 0.143  -  1.780 (0.788 – 4.023), 0.165 |
| **Histology**  Non-SCC | 32 (71.1) | 13 (28.9) | **0.508 (0.279 – 0.925), 0.027** | 0.649 (0.339 – 1.242), 0.192 |
| **Staging Group**  3 – 4 | 69 (51.5) | 65 (48.5) | 1.882 (0.967 – 3.665), 0.063 | - |
| **Baseline mean eGFR (ml/min/1.73m^2^ )** | - | - | 0.998 (0.982 – 1.014), 0.849 | **-** |
| **Baseline mean SCr (mg/dL)** | - | - | 1.281 (0.314 – 5.213), 0.729 | **-** |
| **rs316019 A Carriage** | 18 (50.0) | 18 (50.0) | 1.386 (0.815 – 2.356), 0.228 | - |
| **rs1051740 T Carriage** | 73 (56.2) | 57 (43.8) | 0.883 (0.520 – 1.502), 0.649 | - |
| **rs11615 A Carriage** | 53 (59.6) | 36 (40.4) | 0.845 (0.537 – 1.330), 0.468 | - |
| **rs3212986 A Carriage** | 46 (52.9) | 41 (47.1) | 1.117 (0.708 – 1.760), 0.633 | - |
| **rs13181 G Carriage** | 14 (37.8) | 23 (62.2) | **2.080 (1.270 – 3.407), 0.004** | 1.837 (0.918 – 3.674), 0.086 |
| **rs1799793 T Carriage** | 9 (33.3) | 18 (66.7) | **2.107 (1.238 – 3.586), 0.006** | 1.029 (0.477 – 2.220), 0.940 |

***Reviewer #5:***

Discussion:
A) While the discussion is comprehensive, consider further condensing to maintain focus on the key findings.

**Response to the Reviewer # 5:**

Thank you for your comment. We have revised the “Discussion” part to condense and focus on the findings where applicable on Page 25 - Lines 586-596, Page 27 - Lines 642-657, Page 28 - Lines 659-672 and as follows:

Page 25 - Lines 586-596: “No consensus has been reached regarding SNPs associated with CIN, partly due to significant variations in minor allele frequencies across populations. Previous studies have shown association of these 6 selected SNPS with CIN using AKI as the endpoint. Notably, the minor allele frequency of *EPHX1* rs1051740 differs in the Thai population compared to other populations [36]. Most commonly the reference allele is T, whose expression as homozygous TT genotype is associated with faster metabolism than CC. However, in Thai population, T is the minor allele, with a frequency of 0.49. The *EPHX1* rs1051740 (c.337T>C), is one of the 2 widely studied polymorphisms of the *EPHX1* gene. This variant results in a tyrosine-to- histidine substitution at position 113, leading to a 50% reduction in enzyme activity [37]. Decreased enzymatic activity may reduce the detoxification, increasing the formation of highly reactive metabolites, making the cells more susceptible to challenges.”

Pages 27-28 - Lines 642-657: “Since AKD by itself can lead to poor OS in cancer patients, partly due to the resulting inadequate treatment, we further investigated the effect of these candidate SNPs on OS. In our study, although the OS analysis of AKD vs non-AKD groups was not statistically significant, we observed a trend indicating that patients without AKD had numerically longer OS compared to those with AKD. OS analysis for each selected SNP revealed no association with polymorphisms in *SLC22A2*, *EPHX1* and *ERCC1* genes. However, using a dominant genetic model, OS was associated with *ERCC2* rs13181 and rs1799793: patients with *ERCC2* rs13181-TT genotype and rs1799793-CC genotype exhibited better OS. Notably, these *ERCC2* SNPs showed no association with AKD, suggesting their effects on OS are independent of AKD status. Previously, data on survival in relation to *ERCC2* gene polymorphisms have varied across different populations and cancer types, limiting their application in clinical practice. Variant genotypes of *ERCC2* rs13181 and rs1799793 have been significantly associated with poor survival in Chinese NSCLC patients [49]. A meta-analysis by Yang et al. found that the rs13181 variant G and rs1799793 variant T were poorly associated with OS in NSCLC patients [50]. Among Caucasian gastric cancer patients, rs13181 was not associated with OS, whereas the rs1799793 homozygous variant AA genotype was significantly linked to poor OS [51].”

Page 28 - Lines 659-672: “Stratification of OS analysis by cancer type revealed that both SNPs of *ERCC2* were associated only with HNSCC patients in our cohort under a dominant genetic model. Our findings align with recent studies reporting worse OS for HNSCC patients with the *ERCC2* rs13181 homozygous minor genotype and significantly better OS for those with the rs1799793 homozygous major genotype under the same model [52, 53]. OS stratification for lung cancer patients showed no significant associations in our study. However, an Asian lung cancer study recently reported that *ERCC2* rs13181 TG and TG+GG genotypes were significantly linked to worse OS under a dominant model [54]. These differing results may stem from the small sample size in our lung cancer cohort. While our findings showed no OS association with *ERCC1* rs11615 and rs3212986, these SNPs have been widely studied in various cancers with varying survival outcomes. The G allele of rs11615has been significantly associated with better OS, and the A allele of rs3212986 has been linked to improved PFS in a lung cancer study [55]. Conversely, a meta-analysis by Yang et al. found that the *ERCC1* rs11615 T allele was associated with poor OS in NSCLC patients [50].”

***Reviewer #5:***

*B) Address the implications of the findings for clinical practice, particularly regarding personalized chemotherapy approaches*

**Response to the Reviewer # 5:**

Thank you for your suggestion. We have revised the relevant part on Page 29 - Lines 695-696 and as follows:

“The AKD-associated SNPs can be utilized to tailor treatment protocols for patients undergoing cisplatin-based therapy, advancing personalized chemotherapy approaches Table 5.”

***Reviewer #5:***

*Study Limitations:
The authors acknowledge the study's limitations, including geographic and ethnic constraints. Emphasizing the need for validation in diverse cohorts would strengthen the discussion*

**Response to the Reviewer # 5:**

Thank you for your kind suggestion. We have revised the relevant part to emphasize the need for validation in diverse cohorts on Page 29 – Line 682-685 and subsequently on Page 29 – Line 696-698 as follows:

Page 29 – Lines 682-685: “Additionally, the small sample size, single-institution retrospective study design, and limited number of SNPs, all of which are common in Thais, may restrict the generalizability of our findings to broader populations, highlighting the need for validation in larger and more diverse cohorts.”

Page 29 – Lines 696-698: “Larger prospective studies across diverse populations are necessary to validate these genetic markers for personalized treatment approaches.”

***Reviewer #5:***

*Ethical and Publication Concerns:
Ethical Compliance:
The manuscript states approval from the Human Research Ethics Committee and adherence to the Declaration of Helsinki. This is sufficient, though explicit mention of informed consent obtained for SNP analysis would reinforce compliance.*

**Response to the Reviewer # 5:**

The informed consent for the Ramathibodi Comprehensive Tumor Biobank is a broad consent for future research. If the research is related to genetic studies, patients can choose whether or not to be informed of the results.

***Reviewer #5:***

Publication Ethics:
There are no indications of dual publication or ethical concerns.
Recommendations for Improvement:
Include a concise abbreviation list in the manuscript's main body to improve readability.

**Response to the Reviewer # 5:**

Thank you very much for your kind suggestion, we have gathered all the relevant abbreviations used in the manuscript and listed them on Page 3, Lines: 63 – 84 and as follows:

**“List of Abbreviations**

**CIN:** Cisplatin Induced Nephrotoxicity

**AKI:** Acute Kidney Injury

**AKD:** Acute Kidney Disease

**SNP:** Single Nucleotide Polymorphisms

***OCT2*:** Organic Cation Transporter 2

***SLC22A2*:** Solute Carrier Family 22 member 2

***ERCC1***: Excision Repair Cross-Complementing 1

***ERCC2***: Excision Repair Cross-Complementing 2

***EPHX1:*** Epoxide Hydrolase 1

***XPD:*** Xeroderma Pigmentosum group D

**DME:** Drug Metabolizing Enzyme

**PFS:** Progression-Free Survival

**OS:** Overall Survival

**CTCAE-AKI:** Common Criteria Terminology for Adverse Events - Acute Kidney Injury

**KDIGO:** Kidney Disease Improving Global Outcomes

**ICD-10:** 10^th^ Revision of International Classification of Diseases

**T-Rex:** Thai Reference Exome Database

**MAF:** Minor Allele Frequency

**FDR:** False Discovery Rate

**BH-FDR:** Benjamini-Hochberg False Discovery Rate

**HWE:** Hardy-Weinberg Equilibrium”

***Reviewer #5:***

*Ensure that all cited data is either included in the manuscript or accessible through provided links.*

**Response to the Reviewer # 5:**

No data from external databases were used in this study.

***Reviewer #5:***

*Provide a summary table of key findings with clinical implications to improve accessibility for non-specialist readers.*

**Response to the Reviewer # 5:**

Thank you for your suggestion, we have added a table summarizing the key findings and their potential clinical implications on Page 30 as well as here:

**“Table 5. Summary of key findings and their clinical implications”**

| **Key Findings** | **Potential clinical implications** |
| --- | --- |
| ***EPHX1***  rs1051740 | A tailored treatment protocol to reduce potential AKD occurrence (personalized chemotherapy approach) |
| ***ERCC2***  rs13181  rs1799793 | Prognostic biomarkers for HNSCC patients |

We believe that these revisions based on the reviewers’ comments have substantially improved the quality of our manuscript. In addition to addressing the specific comments raised by the reviewers, we have revised some parts for better clarity.

We hope that the revised version of our manuscript, along with our detailed responses, can clearly address the comments from the editor and reviewers and are sufficient to make our manuscript suitable for publication in PLOS ONE.

Again, thank you very much for the opportunity to revise and resubmit our manuscript to PLOS ONE. We look forward to receiving your reply.

Sincerely,

Natini Jinawath, MD, PhD

Corresponding author
