# Supplementary material for: EPHX1 and ERCC2 polymorphisms are associated with cisplatin-induced nephrotoxicity and prognosis in Thai cancer patients
Source: PLoS One. 2025 Jun 17;20(6):e0324699. doi: 10.1371/journal.pone.0324699 (PMC12173183; doi:10.1371/journal.pone.0324699)
Supplement: Attachment — Submitted filename: Response_to_Reviewers_auresp_3.docx [file pone.0324699.s016.docx]

Dear the Editor in Chief of PLOS ONE,

Thank you for your kind consideration of our manuscript entitled “*EPHX1* and *ERCC2* polymorphisms are associated with cisplatin induced nephrotoxicity and prognosis in Thai cancer patients” (Manuscript Number: **PONE-D-24-18947R2**). We appreciate the time and effort you and the reviewers invested in evaluating our work. We have carefully considered the reviewers’ comments and made significant revisions to address their concerns. Below, we outline our responses to each point raised by the reviewers. All corresponding changes that have been added to the manuscript are marked with “Track Changes”.

**Reviewer’s Comments:**

***Reviewer #6:***

1) Table 2 (L404) presents a comparison of allele frequencies between the T-REx database and the current study (based on the frequencies observed in non-AKD patients). Additionally, for better comparison, global MAF frequencies should be included in the table.

**Response to the Reviewer # 6:**

Thank you for your valuable suggestion. Adding global MAF would certainly provide a better comparison. we have added the revised Table 2 on Page 16 and here:

| **Gene** | **SNP** | **SNP Information** | **Global Frequencies** | | **T-REx Frequencies** | | **Data Allele Frequencies** | | **HWE *P* value** |
| --- | --- | --- | --- | --- | --- | --- | --- | --- | --- |
| ***SLC22A2*** | **rs316019** | Missense c.808G>T (p.270Ala > Ser) | C | A | C | A | C | A | 0.187 |
|  |  |  | 0.895 | 0.104 | 0.890 | 0.11 | 0.8289 | 0.1711 |  |
| ***EPXH1*** | **rs1051740** | Missense c.337T>C (p.Tyr113His) | C | T | C | T | C | T | 0.331 |
|  |  |  | 0.295 | 0.704 | 0.504 | 0.496 | 0.5036 | 0.4964 |  |
| ***ERCC1*** | **rs11615** | Synonymous c.354T>C (p.Asn118Asn) | G | A | G | A | G | A | 0.482 |
|  |  |  | 0.414 | 0.585 | 0.697 | 0.303 | 0.6 | 0.4 |  |
| ***ERCC1*** | **rs3212986** | Synonymous 3’ UTR c*197G>T | C | A | C | A | C | A | 0.261 |
|  |  |  | 0.745 | 0.254 | 0.664 | 0.336 | 0.7205 | 0.2795 |  |
| ***ERCC2*** | **rs13181** | Missense c.2251A>C (p.751lys > Gln) | T | G | T | G | T | G | 0.91 |
|  |  |  | 0.642 | 0.000 | 0.901 | 0.099 | 0.9108 | 0.0892 |  |
| ***ERCC2*** | **rs1799793** | Missense c.934G>A (p.Asp312Asn) | C | T | C | T | C | T | 0.889 |
|  |  |  | 0.684 | 0.315 | 0.937 | 0.063 | 0.9422 | 0.0578 |  |

Global frequencies were obtained from dbSNP (build 157), accessed on 13^th^ March 2025.

The following sentences “Allele frequencies in Thai and global populations were respectively obtained from the Thai Reference Exome Database (T-REx), which contains data from 1,092 Thai individuals (accessed on 25^th^ February 2022), and from dbSNP (build 157) (accessed on 13^th^ March 2025) & “Allele frequencies were obtained from dbSNP (build 157; <https://www.ncbi.nlm.nih.gov/snp/>)**,** and from the T-REx database [30]. are also added in the Materials and Methods (Line 248-251, pages 9-10) and table legend (Line 405-406, page 16), respectively.

2) False Discovery Rate (FDR) was used for multiple comparisons, which is commonly applied in large-scale studies such as GWAS. However, given the small sample size and a limited number of comparisons (169 samples and 6 SNPs), Bonferroni correction is preferable to minimize false positives. In this case, a more balanced approach could be the Holm correction, which is less conservative than Bonferroni but provides better Type I error control than FDR.

**Response to the Reviewer # 6:**

Thank you for your insightful comment. We have performed the Holm-Bonferroni correction for our main results and revised Table 3 accordingly in the manuscript and here as follows:

| **SNP** | **Model** | **Genotype** | **AKD** | **Non-AKD** | **Unadjusted OR**  **(95% CI)** | ***P* value** | **BH-FDR**  ***P* value** | **Holm-Bonferroni**  ***P* value** | **Adjusted OR**  **(95% CI)** | ***P* value Adj** | **BH-FDR P value Adj** | **Holm-Bonferroni *P* Value Adj** |
| --- | --- | --- | --- | --- | --- | --- | --- | --- | --- | --- | --- | --- |
| ***SLC22A2* rs316019** | Co-dominant | CC | 32 (74.4) | 101 (80.1) | 1 |  |  |  | 1 |  |  |  |
|  |  | AC | 11 (25.6) | 22 (17.5) | 1.578 (0.691 - 3.603) | 0.279 | 0.721 | 0.003 | 1.532 (0.656 - 3.578) | 0.323 | 0.738 | 0.003 |
|  |  | AA | 0 (0.0) | 3 (2.4) | - | - | - |  | - | - | - |  |
|  | Dominant | AA+AC | 11 (25.6) | 25 (19.8) | 1.388 (0.615 - 3.131) | 0.429 | 0.881 | 0.003 | 1.362 (0.591 - 3.139) | 0.468 | 0.881 | 0.003 |
|  | Recessive | CC+AC | 43 (100) | 123 (97.6) | 1 |  |  |  | 1 |  |  |  |
|  |  | AA | 0 (0.0) | 3 (2.4) | - | - | - |  | - | - | - |  |
|  | Over-dominant | CC+AA | 32 (74.4) | 104 (82.5) | 1 |  |  |  | 1 |  |  |  |
|  |  | AC | 11 (25.6) | 22 (17.5) | 1.625 (0.712 - 3.708) | 0.249 | 0.693 | 0.002 | 1.576 (0.675 - 3.679) | 0.293 | 0.721 | 0.003 |
|  | Log-Additive |  |  |  | 1.165 (0.562 - 2.415) | 0.681 | 0.935 | 0.005 | 1.157 (0.546 - 2.452) | 0.702 | 0.935 | 0.006 |
| ***EPHX1* rs1051740** | Co-dominant | CC | 6 (14.0) | 33 (26.2) | 1 |  |  |  | 1 |  |  |  |
|  |  | TC | 30 (69.8) | 57 (45.2) | **2.894 (1.091 - 7.680)** | **0.033*** | 0.526 | 0.002 | **3.354 (1.230 - 9.148)** | **0.018*** | 0.384 | 0.002 |
|  |  | TT | 7 (16.2) | 36 (28.6) | 1.069 (0.325 - 3.509) | 0.912 | 0.941 | 0.017 | 1.138 (0.336 - 3.843) | 0.835 | 0.941 | 0.010 |
|  | Dominant | TT+TC | 37 (86.0) | 93 (73.8) | 2.188 (0.846 - 5.655) | 0.106 | 0.526 | 0.002 | 2.459 (0.931 - 6.493) | 0.069 | 0.526 | 0.002 |
|  | Recessive | CC+TC | 36 (83.7) | 90 (71.4) | 1 |  |  |  | 1 |  |  |  |
|  |  | TT | 7 (16.3) | 36 (28.6) | 0.486 (0.198 - 1.192) | 0.115 | 0.526 | 0.002 | 0.475 (0.188 - 1.199) | 0.115 | 0.526 | 0.002 |
|  | Over-dominant | CC+TT | 13 (30.2) | 69 (54.8) | 1 |  |  |  | 1 |  |  |  |
|  |  | TC | 30 (69.8) | 57 (45.2) | **2.793 (1.333 - 5.851)** | **0.006*** | 0.192 | 0.002 | **3.137 (1.450 - 6.785)** | **0.004*** | 0.192 | 0.002 |
|  | Log-Additive |  |  |  | 0.998 (0.607 - 1.642) | 0.996 | 0.996 | 0.050 | 1.039 (0.624 - 1.730) | 0.881 | 0.941 | 0.017 |
| ***ERCC1* rs11615** | Co-dominant | GG | 21 (48.8) | 59 (46.8) | 1 |  |  |  | 1 |  |  |  |
|  |  | AG | 17 (39.5) | 57 (45.2) | 0.837 (0.401 - 1.748) | 0.638 | 0.935 | 0.004 | 0.853 (0.401 - 1.815) | 0.680 | 0.935 | 0.005 |
|  |  | AA | 5 (11.6) | 10 (8.0) | 1.404 (0.430 - 4.587) | 0.574 | 0.935 | 0.004 | 1.081 (0.315 - 3.706) | 0.900 | 0.941 | 0.025 |
|  | Dominant | AA+AG | 22 (51.2) | 67 (53.2) | 0.922 (0.461 - 1.844) | 0.820 | 0.941 | 0.008 | 0.892 (0.437 - 1.821) | 0.755 | 0.935 | 0.007 |
|  | Recessive | GG+AG | 38 (88.4) | 116 (92.1) | 1 |  |  |  | 1 |  |  |  |
|  |  | AA | 5 (11.6) | 10 (7.9) | 1.526 (0.490 - 4.745) | 0.465 | 0.881 | 0.003 | 1.164 (0.357 - 3.796) | 0.800 | 0.941 | 0.008 |
|  | Over-dominant | GG+AA | 26 (60.5) | 69 (54.8) | 1 |  |  |  | 1 |  |  |  |
|  |  | AG | 17 (39.5) | 57 (45.2) | 0.791 (0.391 - 1.601) | 0.516 | 0.917 | 0.004 | 0.841 (0.407 - 1.737) | 0.642 | 0.935 | 0.004 |
|  | Log-Additive |  |  |  | 1.041 (0.609 - 1.779) | 0.883 | 0.941 | 0.013 | 0.966 (0.559 - 1.667) | 0.901 | 0.941 | 0.050 |
|  |  |  |  |  |  |  |  |  |  |  |  |  |

OR, Odds Ratio. 95% CI, 95% Confidence Interval. BH-FDR, Benjamini-Hochberg False Discovery Rate. Adj, adjusted *P* value. * Statistically significant *P* value < 0.05.

| **SNP** | **Model** | **Genotype** | **AKD** | **Non-AKD** | **Unadjusted OR**  **(95% CI)** | ***P* value** | **BH-FDR**  ***P* value** | **Holm- Bonferroni**  ***P* value** | **Adjusted OR**  **(95% CI)** | ***P* value Adj** | **BH-FDR**  ***P* value Adj** | **Holm-Bonferroni**  ***P* value Adj** |
| --- | --- | --- | --- | --- | --- | --- | --- | --- | --- | --- | --- | --- |
| ***ERCC1* rs3212986** | Co-dominant | CC | 20 (46.5) | 62 (49.2) | 1 |  |  |  | 1 |  |  |  |
|  |  | CA | 17 (39.5) | 49 (38.8) | 1.075 (0.509 - 2.270) | 0.849 | 0.941 | 0.010 | 1.136 (0.526 - 2.451) | 0.744 | 0.935 | 0.006 |
|  |  | AA | 6 (14.0) | 15 (12.0) | 1.24 (0.424 - 3.624) | 0.694 | 0.935 | 0.006 | 1.329 (0.442 - 3.988) | 0.612 | 0.935 | 0.004 |
|  | Dominant | AA+AC | 23 (53.5) | 64 (50.8) | 1.114 (0.556 - 2.229) | 0.760 | 0.935 | 0.007 | 1.182 (0.580 - 2.408) | 0.645 | 0.935 | 0.004 |
|  | Recessive | CC+CA | 37 (86.0) | 111 (88.1) | 1 |  |  |  | 1 |  |  |  |
|  |  | AA | 6 (14.0) | 15 (11.9) | 1.2 (0.433 - 3.318) | 0.725 | 0.935 | 0.006 | 1.256 (0.442 - 3.569) | 0.668 | 0.935 | 0.005 |
|  | Over-dominant | CC+AA | 26 (60.5) | 77 (61.1) | 1 |  |  |  | 1 |  |  |  |
|  |  | CA | 17 (39.5) | 49 (38.9) | 1.027 (0.505 - 2.086) | 0.940 | 0.955 | 0.025 | 1.071 (0.516 - 2.222) | 0.853 | 0.941 | 0.013 |
|  | Log-Additive |  |  |  | 1.103 (0.671 - 1.810) | 0.668 | 0.935 | 0.005 | 1.148 (0.692 - 1.905) | 0.592 | 0.935 | 0.003 |
| ***ERCC2* rs13181** | Co-dominant | TT | 37 (86.0) | 95 (75.4) | 1 |  |  |  | 1 |  |  |  |
|  |  | TG | 6 (14.0) | 29 (23.0) | 0.531 (0.203 - 1.384) | 0.195 | 0.594 | 0.002 | 0.470 (0.171 - 1.291) | 0.143 | 0.551 | 0.002 |
|  |  | GG | 0 (0.0) | 2 (1.6) | - | - | - |  | - | - | - |  |
|  | Dominant | GG+TG | 6 (14.0) | 31 (24.6) | 0.496 (0.191 - 1.288) | 0.150 | 0.551 | 0.002 | 0.443 (0.162 - 1.210) | 0.112 | 0.526 | 0.002 |
|  | Recessive | TT+TG | 43 (100) | 124 (98.4) | 1 |  |  |  | 1 |  |  |  |
|  |  | GG | 0 (0.0) | 2 (1.6) | - | - | - |  | - | - | - |  |
|  | Over-dominant | TT+GG | 37 (86.1) | 97 (77.0) | 1 |  |  |  | 1 |  |  |  |
|  |  | TG | 6 (13.9) | 29 (23.0) | 0.542 (0.208 - 1.412) | 0.210 | 0.611 | 0.002 | 0.481 (0.175 - 1.319) | 0.155 | 0.551 | 0.002 |
|  | Log-Additive |  |  |  | 0.491 (0.196 - 1.230) | 0.129 | 0.55 | 0.002 | 0.441 (0.166 - 1.167) | 0.099 | 0.526 | 0.002 |
| ***ERCC2* rs1799793** | Co-dominant | CC | 39 (90.7) | 103 (81.7) | 1 |  |  |  | 1 |  |  |  |
|  |  | CT | 3 (7.0) | 22 (17.5) | 0.360 (0.103 - 1.271) | 0.113 | 0.526 | 0.002 | 0.340 (0.091 - 1.261) | 0.107 | 0.526 | 0.002 |
|  |  | TT | 1 (2.3) | 1 (0.8) | 2.641 (0.161 - 43.265) | 0.496 | 0.907 | 0.003 | 4.056 (0.226 - 72.726) | 0.342 | 0.742 | 0.003 |
|  | Dominant | TT+CT | 4 (9.3) | 23 (18.3) | 0.459 (0.149 - 1.413) | 0.175 | 0.586 | 0.002 | 0.451 (0.140 - 1.453) | 0.183 | 0.586 | 0.002 |
|  | Recessive | CC+CT | 42 (97.7) | 125 (99.2) | 1 |  |  |  | 1 |  |  |  |
|  |  | TT | 1 (2.3) | 1 (0.8) | 2.976 (0.182 - 48.638) | 0.444 | 0.881 | 0.003 | 4.786 (0.265 - 86.183) | 0.288 | 0.721 | 0.002 |
|  | Over-dominant | CC+TT | 40 (93.0) | 104 (82.5) | 1 |  |  |  | 1 |  |  |  |
|  |  | CT | 3 (7.0) | 22 (17.5) | 0.354 (0.100 - 1.250) | 0.107 | 0.526 | 0.002 | 0.331 (0.089 - 1.224) | 0.288 | 0.526 | 0.002 |
|  | Log-Additive |  |  |  | 0.600 (0.225 - 1.599) | 0.308 | 0.73 | 0.003 | 0.609 (0.217 - 1.712) | 0.348 | 0.742 | 0.003 |
|  |  |  |  |  |  |  |  |  |  |  |  |  |

OR, Odds Ratio. 95% CI, 95% Confidence Interval. BH-FDR, Benjamini-Hochberg False Discovery Rate. Adj, adjusted *P* value. * Statistically significant *P* value < 0.05.

These sentences “Multiple comparisons were corrected by Benjamini-Hochberg false discovery rate (FDR) corrected *P*-values, and additionally with Holm-Bonferroni correction” & “although the associations were no longer significant after FDR and Holm-Bonferroni corrections” were also added in the Materials and Methods (Line 296-297, page 11) and Results (Line 421-422, page 17), respectively.

3) Since the researchers applied five genetic models (codominant, dominant, recessive, overdominant, and additive), power analysis was conducted for each of them. However, the additive model is the most justified approach, as it is widely used in SNP association studies due to its general applicability and suitability for small sample sizes. Given the study parameters (n = 169, 6 SNPs), the additive model remains the optimal choice for power calculations, as it accounts for all possible genotypes and provides better sensitivity. However, if the minor allele frequency is particularly low, the dominant model should also be considered.

**Response to the Reviewer # 6**

Thank you for your suggestion. Previously, we have calculated the power for the overall study regardless of the genetic model (Lines 283 - 286, Page 11). As recommended, we have performed power calculation for additive and dominant model. Details of genetic model power calculation were added in the manuscript (Lines 291 - 294, Page 11) and here. Results varied across the two models, 53% being the highest. While it is below the conventional threshold of 80%, suggesting limited statistical power to detect significant association due to small size, we acknowledged this as a study limitation in the manuscript (Lines 686 - 689, Page 29) and here.

Lines 283 - 286, Page 11: “Power calculations showed that a sample size of 169 patients, assuming 25% nephrotoxicity, would provide our study with 80% power to identify a statistically significant association between the selected SNPs and AKD, with an alpha of 0.05 and an odds ratio of 2.8.

Lines 291 - 294, Page 11 “For the additive and dominant genetic models, power calculations were conducted using STATA 14, power two proportions and RStudio 4.4.2 *genpwr* package, respectively. For the six SNPs analyzed, the estimated power varied with 53% being the highest”.

Lines 686 - 689, Page 29: “Additionally, the small sample size, single-institution retrospective study design, and limited number of SNPs, all of which are common in Thais, may restrict the generalizability of our findings to broader populations, highlighting the need for validation in larger and more diverse cohorts”.

4) L482 (R2): The TC genotype of EPHX1 rs1051740 was significantly associated with an increased risk of AKD in the codominant model [OR 13.333, 95% CI 1.684 - 105.533, P = 0.014].
The confidence interval (CI) of 1.684 - 105.533 is extremely wide, indicating high uncertainty (low precision) in the effect estimation. This result cannot be considered reliable. Its interpretation should be approached with caution, as the large uncertainty makes the odds ratio (OR) estimate questionable. It is essential to check for the potential influence of outliers and conduct bootstrap estimation of the CI to assess its stability.

**Response to the Reviewer # 6**

**Table 4.1: Genotype frequency of EPHX1 rs1051740 under the codominant model in the HNSCC-only cohort**

| **Codominant model** | **No AKD** | **AKD** | **Total** |
| --- | --- | --- | --- |
|  |  |  |  |
| **CC** | 26 (96.3) | 1 (3.7) | 27 (100) |
| **TC** | 39 (66.1) | 20 (33.9) | 59 (100) |
| **TT** | 29 (82.86) | 6 (17.14) | 35 (100) |
| **Total** | 94 (77.69) | 27 (22.31) | 121 (100) |
|  |  |  |  |

We appreciate your observations regarding wide confidence interval (CI) of SNP rs1051740 and the reliability of this result.

Comparing to the combined three-cancer-type cohort, the sample size of our head and neck cancer-only cohort is smaller, and only has one case with the “CC” EPHX1 rs1051740 genotype (Table 4.1). This by itself could lead to the overestimation of results and contribute to a wider CI. To address this concern, we performed a bootstrap analysis with 1,000 repetitions. The bootstrap estimates, including the odds ratio (OR) and CI, are now included in the updated Supplementary Table S4A. Also, we added the sentences here: “Of note, bootstrap estimation was performed for the head and neck cancer-only cohort to correct for the wide confidence interval, which could lead to overestimation of the results S4A Table”. & “Our association analysis results show *EPHX1* rs1051740 heterozygous genotype to be associated with AKD in our combined three-cancer-type cohort. This SNP was also significantly associated with AKD in the head and neck cancer-only cohort, although the confidence interval (CI) was wide, likely due to the smaller sample size” in our Results (Lines 489-491, Page 22) & Discussion (Lines 611 - 614, Page 26), respectively.

Additionally, we conducted a comparison between 1,000 and 10,000 bootstrap repetitions for this specific SNP. We found that the effect size (OR) remained consistent across both bootstrap iterations, and the improved confidence intervals were comparable when both repetitions were

used (Table 4.2). Based on these findings, we opted to use 1,000 bootstrap repetitions for all other SNPs and models as shown in the revised Supplementary Table S4A.

Furthermore, the model demonstrated good reliability and validity, with an AUC of 0.77 (Figure 4.1) and a non-significant Hosmer-Lemeshow test (p = 0.571) (Table 4.3) after adjusting for age and sex. These results suggest a good model fit and support the robustness of our findings.

**Table 4.2: Bootstrap estimation comparison between 1,000 and 10,000 repetitions in EPHX1 rs1051740 co-dominant model in HNSCC-only cohort.**

|  | | | **Bootstrap estimation repetitions** | | | |
| --- | --- | --- | --- | --- | --- | --- |
| **Co-dominant** | **AKD** | **Non-AKD** | **1,000 repetitions** | ***P value*** | **10,000 repetitions** | ***P value*** |
| CC | 1 (3.7) | 26 (27.7) | 1 |  | 1 |  |
| TC | 20 (74.1) | 39 (41.5) | **17.319 (5.131-58.460)** | **<0.001** | **17.319 (5.131-58.460)** | **<0.001** |
| TT | 6 (22.2) | 29 (30.8) | **5.824 (1.357-24.985)** | **0.018** | **5.824 (1.408 - 24.080)** | **0.015** |

**Figure 4.1: AUC for the EPHX1 rs1051740**

**Table 4.3: Hosmer-Lemeshow test for EPHX1 rs1051740 in HNSCC-only cohort shows a non-significant result**

| Logistic model for AKD event goodness-of-fit test: | |
| --- | --- |
|  |  |
| Number of observations | 121 |
| Number of covariate patterns | 119 |
| Pearson chi2(114) | 110.64 |
| Prob > chi2 | 0.5715 |

5) Were other factors, such as hydration status, concurrent medications, and chemotherapy regimens, considered in the study? Their incomplete accounting could introduce bias into the results.

**Response to the Reviewer # 6:**

Thank you for raising this valid concern. We agree that these factors could introduce bias into the results. However, we acknowledge these as limitations of our study and addressed this in the manuscript on Line 678 – 685, Page 29 and here as follows:

“While this study provides significant insights into the genetic predictors of cisplatin-induced nephrotoxicity, several limitations should be acknowledged. Although we accounted for age and sex in our logistic regression models, other potential confounding factors, such as details of the chemotherapy regimens, pre-chemotherapy hydration status during treatment, and premedication protocols, were not included in the analysis. This is primarily because our study cohort consisted of patients with various tumor types who were treated with different cisplatin-containing regimens, making it challenging to uniformly account for these variables. These factors are known to influence nephrotoxicity outcomes and may have affected our findings. “

We believe that these revisions based on the reviewers’ comments have substantially improved the quality of our manuscript. In addition to addressing the specific comments raised by the reviewers.

We hope that the revised version of our manuscript, along with our detailed responses, can clearly address the comments from the editor and reviewers and are sufficient to make our manuscript finally suitable for publication in PLOS ONE.

Again, thank you very much for the opportunity to revise and resubmit our manuscript to PLOS ONE. We look forward to receiving your reply.

Sincerely,

Natini Jinawath, MD, PhD

Corresponding author
